# Mapping and Functional Characterization of Homologous Genes *AhSUCA06* and *AhSUCA16* Underlying Sucrose, Oil and Protein Contents in Peanut ( *Arachis hypogaea* L.)

**DOI:** 10.1111/pbi.70667

**Published:** 2026-04-18

**Authors:** Yuzhen Zheng, Feiyan Qi, Lei Shi, Ziqi Sun, Hongfei Liu, Hua Liu, Li Qin, Juan Wang, Chenyang Zhi, Mengmeng Wang, Ziqiang Mo, Stefano Pavan, Xiao Wang, Yaojun Hu, Huanhuan Zhao, Xiaona Li, Wenzhao Dong, Meng Zhang, XiaoDong Dai, Zheng Zheng, Xinyou Zhang

**Affiliations:** ^1^ College of Agronomy Shenyang Agricultural University Shenyang China; ^2^ Institute of Crop Molecular Breeding, Henan Academy of Agricultural Sciences/Henan Provincial Key Laboratory for Genetic Improvement of Oil Crops/Key Laboratory of Oil Crops in Huang‐Huai‐Hai Plains, Ministry of Agriculture, Sciences Zhengzhou China; ^3^ The Shennong Laboratory Zhengzhou China; ^4^ National Innovation Centre for Bio‐Breeding Industry Xinxiang China; ^5^ Department of Soil, Plant and Food Sciences University of Bari Aldo Moro Bari Italy

**Keywords:** CRISPR/Cas9, functional molecular markers, homologous genes, peanut, QTL mapping, seed oil content, seed protein content, seed sucrose content

## Abstract

Cultivated peanut ( *Arachis hypogaea* L.) is an important oilseed and cash crop, and seed sucrose content (SSC), seed oil content (SOC) and seed protein content (SPC) are key determinants of seed flavour, texture, and overall quality. Identifying quantitative trait loci (QTLs) and candidate genes associated with SSC, SOC and SPC is therefore of considerable importance for peanut genetics and breeding. In this study, two recombinant inbred line (RIL) populations derived from reciprocal crosses between the lines Jihuatian1 (JHT1) and PI478819 (PI) were used to detect major QTLs for SSC, SOC and SPC through bulked segregant analysis combined with whole‐genome sequencing (BSA‐seq). Multiple lines of evidence supported the homologous gene pair *AhSUCA06* and *AhSUCA16* as candidate genes underlying the epistatic QTLs *qA06.1* and *qA16.1*, which exhibited major and stable effects across multiple phenotypic evaluations. Furthermore, the function of *AhSUCA06* was validated through CRISPR/Cas9‐mediated genetic transformation. Subcellular localization assays using GFP fusion proteins, together with dual‐luciferase reporter assays, demonstrated that *AhSUCA06* and *AhSUCA16*—both containing a DUF7950 domain of previously unknown function—localize to the nucleus and act as transcriptional repressors. In addition, DAP‐seq analysis suggested that these genes may regulate pathways related to glycolysis and gluconeogenesis. Overall, this study provides new insights into the molecular mechanisms underlying the regulation of SSC, SOC and SPC in peanut and offers valuable information to support the genetic improvement of seed quality traits in peanut breeding programs.

## 1 Introduction

Cultivated peanut ( *Arachis hypogaea* L.) is a multipurpose oilseed legume rich in unsaturated fatty acids, high‐quality proteins, arginine, vitamin E and other plant nutrients (Arya et al. 2016; Kris‐Etherton et al. 2008). Notably, it is one of the most widely consumed legume crops worldwide. It is widely cultivated for edible oil production, direct human consumption, and various industrial applications, popular snacks, including chocolate and cookies (Çiftçi and Suna 2022). The primary driver of peanut consumption is its excellent taste, which is largely determined by the seed sucrose content (SSC) (Bishi et al. 2015; Zhang et al. 2023). However, SSC in most peanut varieties is typically lower than 3%, with SSC only a few varieties exceeding 5% (Bishi et al. 2015; Wang et al. 2011). Earlier research indicated that SSC greater than 5% results in a relatively pronounced sweetness and improved taste (Lykomitros et al. 2016). As their key biosynthetic precursor, sucrose regulates the oil and protein contents in peanut kernels (Umretiya et al. 2025). In peanut, SSC is negatively correlated with the seed oil content (SOC), but positively correlated with the seed protein content (SPC) (Huai et al. 2024; Zhi et al. 2024), which determines the flavour and nutritional quality of peanut products (Arya et al. 2016). The consumption of edible peanut oil and food has recently been increasing worldwide (Wang et al. 2024). Therefore, exploring the genetic and molecular mechanisms mediating the regulation of SSC, SOC and SPC may have considerable implications for the molecular breeding of peanut.

SSC, SOC and SPC are complex quantitative traits controlled by multiple genetic factors (Chen et al. 2024; Guo et al. 2023; Huai et al. 2024; Sun et al. 2022; Wang et al. 2024; Yang, Li, et al. 2023). Several studies have mapped quantitative trait loci (QTLs) underlying the genetic control of peanut SSC, SOC and SPC. QTLs identified so far for SSC are primarily located on chromosomes A06, A07, A08 and A16, which encompass genomic regions of 28.26, 1810, 2110 and 700 kb, respectively, and explain from 5.43% to 47.5% of sucrose content variation (Guo et al. 2023; Huai et al. 2024; Li et al. 2023; Wang et al. 2024). Notably, the QTL on chromosome A06 co‐localizes with a QTL for SOC, suggesting possible epistatic effects (Huai et al. 2024). Other QTLs for SOC have been localized on chromosomes A02 (Shasidhar et al. 2017), A05 (Pandey et al. 2014; Sun et al. 2022), A06 (Wilson et al. 2017), A08 (Liu et al. 2020; Yang, Li, et al. 2023), A10 (Shasidhar et al. 2017), B03 (Huang et al. 2015) and B10 (Guo et al. 2021). QTLs controlling SPC were mainly identified through biparental mapping and genome‐wide association studies (Sun et al. 2022; Zhang et al. 2021). A major QTL for SPC was mapped in a 2.3 Mb interval on chromosome A20 on the basis of a QTL‐seq analysis (Chen et al. 2024). However, the substantial physical distances between these loci have hindered the implementation of marker‐assisted selection, restricted the exploration of genetic relationships among SSC, SOC and SPC, and impeded the identification of candidate genes linked to SSC, SOC and SPC. Consequently, the identification and functional validation of genes coordinately regulating SSC, SOC and SPC in peanut will provide valuable insights that may enable breeders to design more precise breeding strategies and develop elite varieties tailored to specific quality requirements.

The application of next‐generation sequencing (NGS) technologies for peanut research (Varshney et al. 2014) and the publication of tetraploid cultivated peanut genome sequences (Bertioli et al. 2019; Chen et al. 2019; Zhuang et al. 2019) as well as the assembly of the cultivated peanut telomere‐to‐telomere genome (Wang et al. 2025) have been conducive to conducting polymorphism‐based molecular marker studies and QTL mapping. For example, BSA‐seq, which integrates a bulked segregant analysis (BSA) and NGS, is a simple and efficient technique that has been used for predicting candidate genomic regions in crops. Previous studies involving peanut identified numerous genetic loci or candidate genes associated with pod size (Yang, Luo, et al. 2023; Zhao et al. 2023), sucrose content (Guo et al. 2023; Huai et al. 2024; Li et al. 2023), and disease resistance (Wu et al. 2024; Yu et al. 2024) on the basis of BSA‐seq combined with linkage mapping. Peanut has a large genome and complex genetic mechanisms regulating SSC, SOC and SPC. Hence, cloning and functionally validating key genes influencing these traits is challenging. To date, there have been no published reports on gene mapping and functional validation in peanut.

To elucidate the molecular mechanisms and regulatory pathways underlying SSC, SOC and SPC in peanut, this study was completed to map the major QTLs associated with these traits, clone key regulatory genes, develop functional markers, and validate target genes. The study findings may provide the theoretical basis for future research on the mechanisms regulating SSC, SOC and SPC in peanut.

## 2 Results

### 2.1 Phenotypic Evaluation of the Mapping Population and the Parental Varieties

The two parent varieties, Jihuatian1 (JHT1) and PI478819 (PI), differed significantly in terms of SSC, SOC and SPC across multiple environments. Specifically, SSC and SPC were significantly higher in JHT1 than in PI, whereas the opposite trend was observed for SOC (Tables 1 and S1). SSC, SOC and SPC ranges for the JHT1 × PI RIL (JP‐RIL) population were 1.98%–7.96%, 45.64%–56.91% and 21.86%–28.50%, respectively (Table 1). Similar ranges were observed for the reciprocal PI × JHT1 RIL (PJ‐RIL) population (Table S1).

**TABLE 1:** Descriptive statistics for the seed sucrose content (SSC), seed oil content (SOC), and seed protein content (SPC) in the two parents and the JHT1 × PI RIL population (JP‐RIL).

Trait	Env	Parents		JP‐RIL				Normality analysis			Sig	*H* ^2^
		JHT1 (%)	PI (%)	Range	Mean	SD	CV (%)	Skew	Kurt	W (*P*)		
SSC	E1	7.74***	2.78	1.36–8.49	4.44	1.79	40.35	0.70	−0.81	0.89 (< 0.001)	***	0.93
	E2	7.44***	2.07	1.18–9.48	4.05	1.65	40.77	0.73	−0.62	0.90 (< 0.001)		
	BLUP	7.36***	2.55	1.98–7.96	4.29	1.52	35.57	0.69	−0.90	0.89 (< 0.001)		
SOC	E1	46.50***	56.79	45.02–58.04	52.76	3.30	6.26	−0.77	−0.64	0.90 (< 0.001)	***	0.93
	E2	45.87***	56.48	42.11–58.75	52.56	3.17	6.04	−0.73	−0.57	0.91 (< 0.001)		
	BLUP	46.63***	56.35	45.64–56.91	52.59	2.86	5.43	−0.73	−0.79	0.89 (< 0.001)		
SPC	E1	28.67***	22.88	20.91–30.90	25.26	2.02	8.01	0.57	−0.56	0.95 (< 0.001)	***	0.88
	E2	27.16***	22.26	19.11–29.94	23.88	2.05	8.58	0.50	−0.52	0.97 (< 0.001)		
	BLUP	27.52***	22.81	21.86–28.50	24.61	1.62	6.60	0.62	−0.73	0.93 (< 0.001)		

*Note:* Sig, significance of the effects of the genotype (G), environment (E), and genotype × environment interaction (G × E) as determined by ANOVA; ***, significant at *p* < 0.001; *H* ^2^, broad‐sense heritability per mean. Abbreviations: BLUP, best linear unbiased prediction; CV, coefficient of variation; Env, environment; Kurt, Kurtosis; Skew, Skewness; w, Shapiro–Wilk value.

Genotype, environment, and genotype × environment interaction significantly influenced all three quality‐related traits under study, as resulting from ANOVA (Tables 1 and S1). Broad‐sense heritability (*H* ^2^) estimates across populations and environments ranged from 0.88 to 0.96, suggesting strong genetic control. Substantial phenotypic variations and transgressive segregation were observed for all traits and both RIL populations, with an approximately bimodal frequency distribution (Figure S1). Accordingly, the Shapiro–Wilk normality test for the two RIL populations in two environments indicated deviation from normality (Table 1 and Table S1). Correlation analyses conducted on the two RIL populations indicated significant negative correlation between SSC and SOC (*r* = −0.95 to −0.97) and between SOC and SPC (*r* = −0.91 to −0.94), and positive correlation between SSC and SPC (*r* = 0.77 to 0.86) (Tables S2 and S3).

### 2.2 Identification of Genomic Regions Regulating SSC, SOC and SPC via BSA‐Seq

A total of 51 RILs with high SSC, high SPC and low SOC and 41 RILs with low SSC, low SPC and high SOC were selected to construct high and low sucrose content bulked pools (HSP and LSP), respectively (Table S4). DNA from these pools and the two RIL parental lines were used for whole‐genome resequencing (WGS), which generated 65.87, 65.11, 98.88 and 90.33 Gb data for JHT1, PI, HSP and LSP, respectively. Sequencing depth and mapping rate ranged from 22.85× to 38.56× and from 97.3%–99.0%, respectively, while the genome coverage rate exceeded 98.72% (Table S5).

**FIGURE 1:**
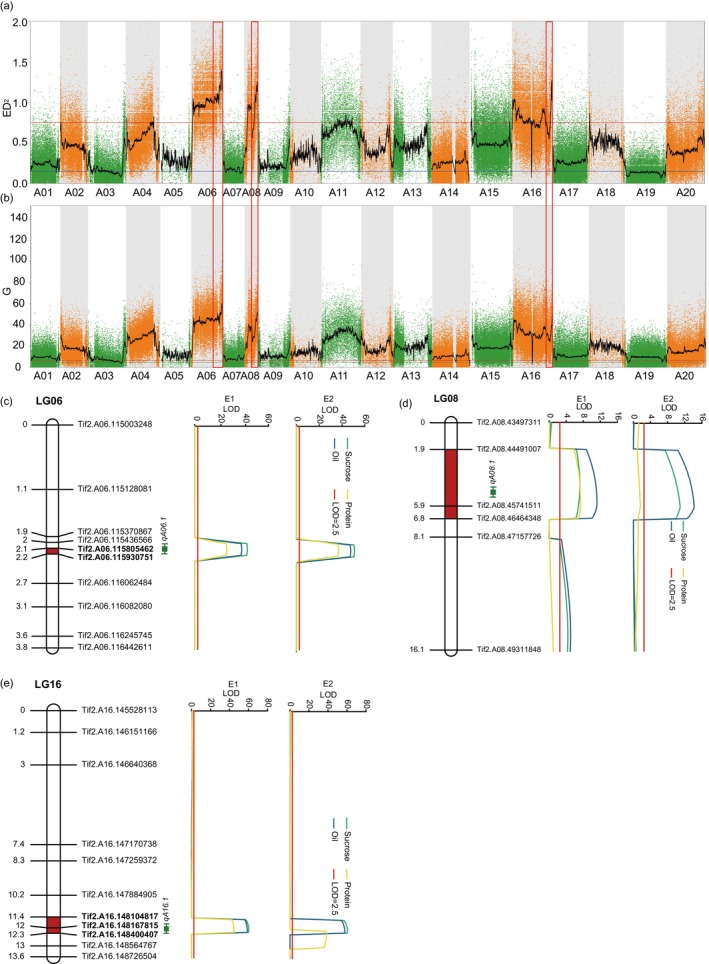
QTLs for SSC, SOC and SPC identified on the basis of BSA‐seq and linkage mapping. Squared ED (a) and G values (b) for chromosomes. (c–e) QTLs and LOD curves for SSC, SOC and SPC in the JP‐RIL population on LG06 (c), LG08 (d) and LG16 (e), with red boxes indicating the candidate region for SSC, SOC and SPC.

A total of 366 023 single nucleotide polymorphisms (SNPs) and insertions/deletions (InDels) that were polymorphic between the two parents were used to identify candidate regions associated with SSC, SOC and SPC on the basis of Δ(SNP‐index), G‐statistic, Fisher's exact test, and ED. Overlapping intervals identified by these four approaches, which were considered as candidate QTL regions for SSC, SOC and SPC, were located on chromosomes A06 (104.86–118.42 Mb), A08 (44.41–51.52 Mb) and A16 (146.43–150.69 Mb) (Table S6; Figure 1a,b; Figure S2a,b).

### 2.3 High‐Resolution Genetic Map Construction, QTL Mapping for SSC, SOC and SPC, and Epistatic QTL Interaction Analysis in Reciprocal RIL Populations

Sequence information on 33 SNP/InDel loci within the three candidate intervals was used to develop kompetitive allele‐specific PCR (KASP) markers (Table S7), which were used to genotype RILs from the JP and PJ RIL populations. In addition, 27 KASP markers were used to construct three linkage groups (LGs). Specifically, in the JP (or PJ) RIL population, 10 (or 10), 11 (or 10) and 6 (or 6) markers were mapped on chromosomes A06, A16 and A08, spanning a genetic distance of 3.8 cM (or 5.7 cM), 13.6 cM (or 12.7 cM) and 16.1 cM (or 17.9 cM), respectively. Three QTLs (*qA06*.1, *qA16.1* and *qA08.1*) for SSC and SOC were consistently detected on LGs corresponding to chromosomes A06, A16 and A08, respectively, across both RIL populations and environments (Table S8). For SPC, only *qA06*.*1*, *qA16*.*1* were stably detected.

The major QTL *qA06*.*1* was located between the KASP markers Tif2.A06.115436566 and Tif2.A06.115930751, and was associated with percentages of variance explained (PVEs) of 24.0%–29.7%, 21.6%–26.6% and 16.9%–32.4% for SSC, SOC and SPC, respectively (Figure 1c; Figure S3a). The major QTL *qA08.1* was located between the KASP markers Tif2.A08.44491007 and Tif2.A08.47157726, with estimated PVEs of 1.38%–4.70% and 3.31%–6.41% for SSC and SOC, respectively (Figure 1d; Figure S3b). Finally, the QTL *qA16.1* was located between the KASP markers Tif2.A16.148104817 and Tif2.A16.148400407, with PVEs of 30.6%–37.7%, 28.0%–37.4% and 18.7%–24.0% for SSC, SOC and SPC, respectively (Figure 1e; Figure S3c).

Epistatic QTLs (Epi‐QTLs) analysis for SSC, SOC and SPC identified two interacting loci. The first, Epi‐QTL1 (*qA06.1*), was located in the A06:115436566–115930751 genomic interval, whereas Epi‐QTL2 (*qA16.1*) was located in the A16:148104817–148400407 genomic interval. LOD values ranged from 40.8 to 50.3 for SSC, 29.4 to 52.1 for SOC, and 9.5 to 27.1 for SPC. The corresponding phenotypic variance explained (PVE) ranged from 72.2% to 79.2% for SSC, 66.6% to 77.4% for SOC, and 49.4% to 64.0% for SPC (Table S9).

### 2.4 Definition of QTL Intervals and Candidate Gene Prediction

**FIGURE 2:**
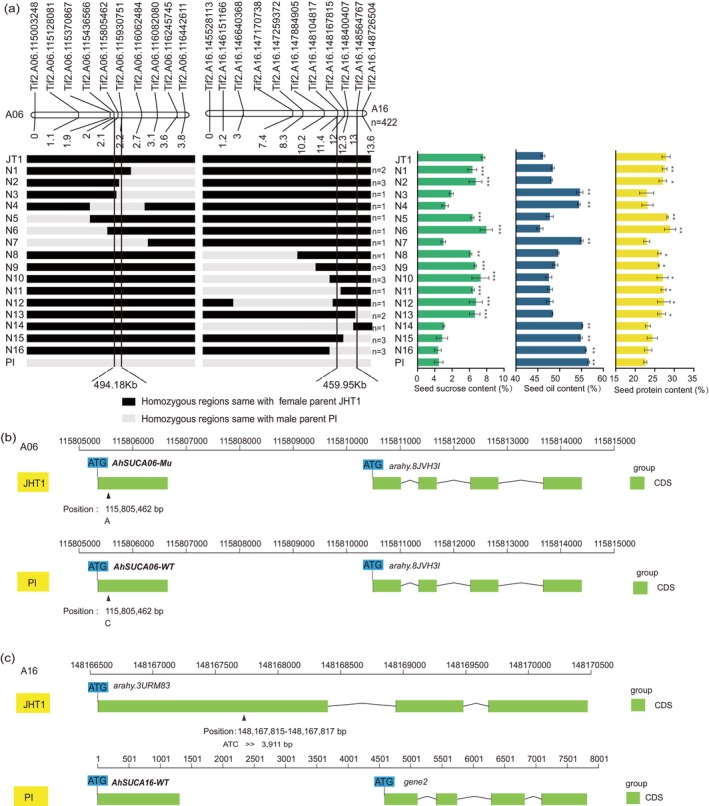
Validation of candidate intervals and candidate genes for *qA06.1* and *qA16.1*. (a) Candidate intervals for *qA06.1* and *qA16.1* according to the genotypes and phenotypes of recombinant lines. (b) and (c) Structure and mutation of *AhSUCA06* and *AhSUCA16*, respectively. In (a), N1–N16 represent recombinant lines classified into 16 distinct types; ‘n’ represents the number of lines corresponding to the recombination type. Data are presented as the mean ± standard deviation of four biological replicates of the JP‐RIL population in 2 years. Significant differences are indicated as follows: *(*p* < 0.05), **(*p* < 0.01), ***(*p* < 0.001) (Student's *t*‐test).

Following the genotyping of JP‐RIL lines, 28 recombinant lines displaying 16 different genotypes (N1–N16) were selected for *qA06.1* and *qA16.1* validation and fine‐mapping (Figure 2a). Based on the genotypic and phenotypic data of N1–N7, *qA06.1* was located between the markers Tif2.A06.115436566 and Tif2.A06.115930751. Lines with the JHT1 genotype (N1, N2, N5 and N6) had high SSC and SPC but low SOC, whereas lines with the PI genotype (N3, N4 and N7) had low SSC and SPC but high SOC. Based on data of lines N8–N16, *qA16.1* was positioned between the markers Tif2.A16.148104817 and Tif2.A16.148564767. Lines with the JHT1 genotype (N8–N13) had high SSC and SPC but low SOC, whereas lines with the PI genotype (N14–N16) had low SSC and SPC but high SOC.

Genomic comparison between the two parents revealed 153 SNPs and InDels within the candidate interval (494.18 kb) for *qA06.1*, of which 27 resulted in exon mutations in 12 genes (Table S10). As for *qA16.1*, 102 SNPs and InDels were identified, of which four resulted in exon mutations in four genes (Table S10). Given the significant epistatic interaction between *qA06.1* and *qA16.1*, suggesting a possible coordinated or redundant function of homologous genes, the two homologous genes *arahy.3URM83* (designated *AhSUCA16*) and *arahy.42CAD1* (designated *AhSUCA06*), located within the *qA16.1* and *qA06.1* QTLs, respectively, were considered strong candidate genes associated with SSC, SOC and SPC and selected for functional characterization. Notably, a stop‐gain C → A mutation was detected in JHT1 *AhSUCA06* (A06:115805462), resulting in the premature termination of protein translation (Figure 2b; Figure S4), and a large deletion, resulting in a frameshift mutation, was detected in JHT1 *AhSUCA16* (Figure 2c). Furthermore, a comparison of *AhSUCA16* sequences in PI and JHT1 revealed a fragment deletion in *AhSUCA16* in JHT1 (Figures S5 and S6).

### 2.5 Development and Validation of Functional Markers for SSC, SOC and SPC

**FIGURE 3:**
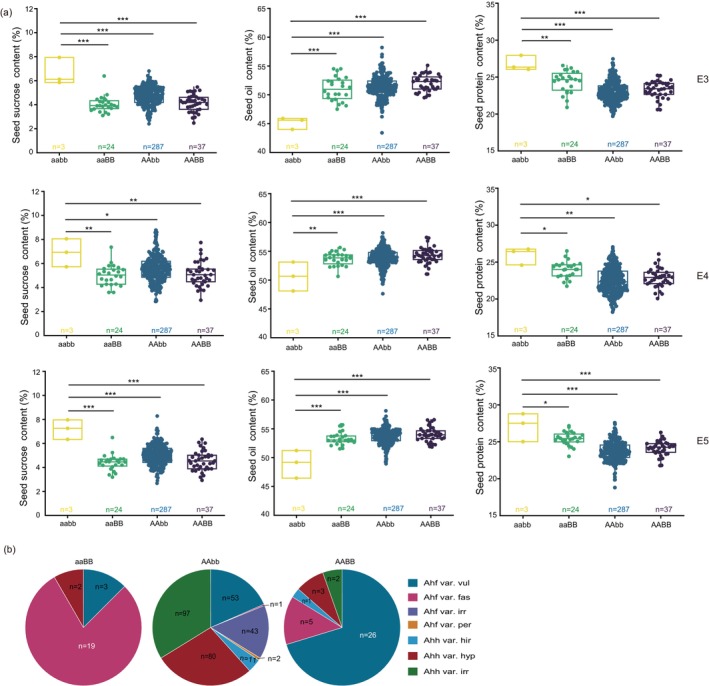
Validation of the genetic effects of KASP markers Tif2.A06.115805462 and Tif2.A16.148167815 associated with a QTL for SSC, SOC and SPC in 353 natural germplasm resources across all environments. (a) Genotypic diversity of Tif2.A06.115805462 and Tif2.A16.148167815 in all environments. (b) Distribution of Tif2.A06.115805462 and Tif2.A16.148167815 polymorphisms in natural germplasm resources. Asterisks indicate significant differences (Student's *t*‐test; *p < 0.05, **p < 0.01, ***p < 0.001).

Two functional markers (Tif2.A06.115805462 and Tif2.A16.148167815) were developed for SSC, SOC and SPC according to the DNA polymorphisms detected in *AhSUCA06* and *AhSUCA16*. The association between these markers and phenotypes was validated across all environments for the JP‐RIL and PJ‐RIL populations and a peanut germplasm panel consisting of 353 accessions. The genotypes for Tif2.A06.115805462 and Tif2.A16.148167815 in JHT1 and PI were designated as aabb and AABB, respectively. Accordingly, the genotypes of RILs and 353 accessions were classified into four categories: aabb, aaBB, AAbb and AABB. SSC and SPC were significantly higher for aabb than in the other three genotype categories, whereas the opposite trend was observed for SOC (Figure 3a; Figure S7a,b). Notably, in the two RIL populations, when the genotypes aaBB and AAbb displayed significantly higher SSC and SPC and lower SOC than the genotype AABB (Figure S7). Therefore, KASP markers Tif2.A06.115805462 and Tif2.A16.148167815 may be useful for analysing SSC, SOC and SPC.

Among the 353 accessions in the natural peanut panel, 287 had the AAbb genotype (Figure 3b), indicating that most peanut germplasm carry a mutated *AhSUCA16* allele. Of the 25 *A. hypogaea* subsp. *fastigiata* var. *fastigiata* (*Ahf*. var. *fas*) accessions, 19 exhibited the aaBB genotype and one exhibited the aabb genotype (Figure 3b), suggesting that the *AhSUCA06* gene is mutated in the majority of *Ahf*. var. *fas* accessions. Among the 37 accessions with the AABB genotype, 26 were *A. hypogaea* subsp. *hypogaea* var. *hirsuta* (*Ahh*. var. *hir*) (Figure 3b), implying that most *Ahh*. var. *hir* accessions have no mutation in either *AhSUCA06* or *AhSUCA16*. The aabb genotype was detected in only three accessions, which were derived from *Ahf*. var. *fas*, *Ahf*. var. *irr* and *Ahh*. var. *irr*, respectively.

### 2.6 Functional Characterization of *AhSUCA06*

**FIGURE 4:**
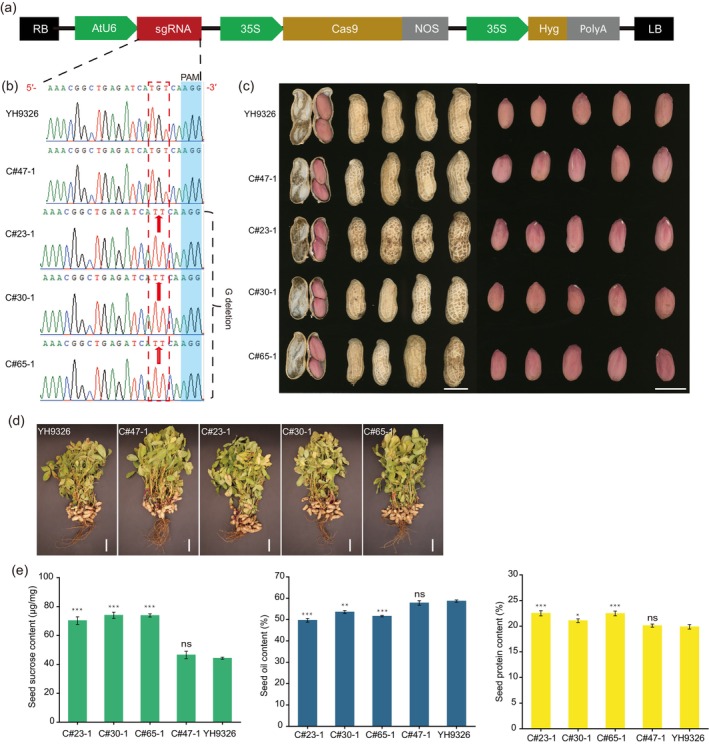
Functional characterization of *AhSUCA06* via CRISPR/Cas9. (a) Schematic diagram of the CRISPR/Cas9 gene‐editing vector. (b) Electropherogram of sanger sequencing results for the *AhSUCA06* sgRNA target site, with base mutation sites highlighted in a red box. (c) Phenotypic traits of mature seeds from C#23–1, C#30–1, C#65‐1, YH9326 and C#47‐1. Scale bars, 2 cm. (d) Morphological characteristics of mutants, WT (YH9326), and negative control plants. Scale bars, 5 cm. (e) SSC, SOC and SPC in mature dry seeds of C#23‐1, C#30‐1, C#65‐1, YH9326 and C#47‐1. Data are presented as the mean ± standard deviation of more than three biological replicates. Asterisks denote statistically significant differences relative to WT (*p* values from Student's *t*‐test; *p < 0.05, ***p < 0.001, ‘ns’ indicates not significant).

A CRISPR/Cas9 expression vector (Figure 4a) was constructed using a single‐guide RNA (sgRNA) designed on the basis of the *AhSUCA06* coding sequence (CDS) and then introduced into the cultivar Yuhua9326 (YH9326) via particle bombardment. A total of 68 callus cultures were generated, of which 48 lines were identified as positive transformants (overall transformation rate of 70.6%) (Table S11). The regeneration of plants yielded 11 T_0_ generation transgenic and two negative control lines (Table S12). Sequencing results indicated that the homozygous mutants were mainly characterized by a G‐deletion corresponding to position 17 of the sgRNA target site (Figure 4b). At maturity, plant and seed morphology of mutant lines (C#23‐1, C#30‐1 and C#65‐1) in the T_1_ generation did not differ significantly compared to wild‐type (WT) YH9326 and the negative control line (C#47‐1) (Figure 4c,d).

Phenotypic evaluation revealed significantly higher SSC and SPC in mature dry seeds of T_1_ mutant lines with respect to the negative control. Conversely, SOC was significantly lower in mutant lines than in the negative control. No significant differences were observed between WT and the negative control (Figure 4e).

### 2.7 Prediction of *AhSUCA06* and *AhSUCA16* Functions

**FIGURE 5:**
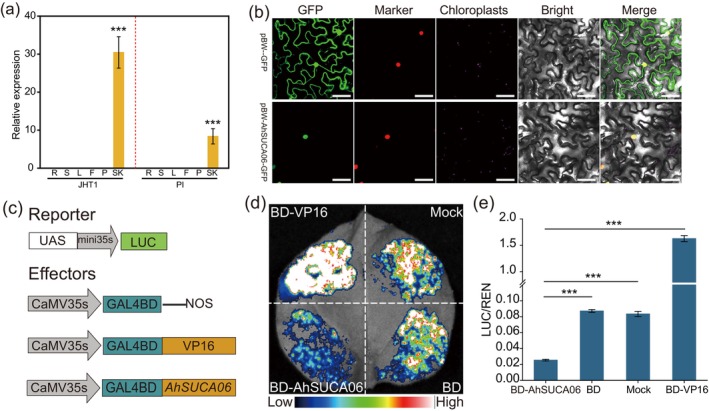
Molecular characterization of *AhSUCA06*. (a) *AhSUCA06* expression levels in various organs revealed by qRT‐PCR. R, root; S, stem; L, leaf; F, flower; P, peg; SK: Seed kernel. Data are presented as the mean ± standard error of three biological replicates. Asterisks indicate significant differences between SK and other organs (Student's *t*‐test; ***p < 0.001). (b) Subcellular localization of *AhSUCA06*. AhSUCA06‐GFP fusion protein was co‐localized with the nuclear marker in tobacco leaf epidermal cells. Scale bars, 50 μm. (c–e) Transcriptional activity analysis of *AhSUCA06* using the DLR assay system. (c) Schematic representation of reporter and effectors. (d) Representative image of a tobacco leaf at 48 h after infiltration. (e) Measurement of relative luciferase activity (LUC/REN). Data are presented as the mean ± standard deviation of three biological replicates. Asterisks indicate significant differences between BD‐AhSUCA06 and the control group (Student's *t*‐test; ***p < 0.001).

To explore the expression patterns of *AhSUCA06* and *AhSUCA16*, qRT‐PCR analysis was performed at 55 days after flowering in the different tissues of JHT1 and PI, including root, stem, leaf, flower, peg and seed. Both *AhSUCA06* and *AhSUCA16* were exclusively expressed in seeds (Figure 5a; Figure S8). Based on the functional annotation available for the *A. hypogaea* cv. Tifrunner v2.0 reference genome, *AhSUCA06* encodes an uncharacterized protein.

To investigate subcellular expression of the AhSUCA06 and AhSUCA16 proteins, fusion constructs comprising these genes and a green fluorescent protein (GFP)‐encoding sequence were transiently co‐expressed with the nuclear marker NLS‐mKate in *N*. *benthamiana* leaves. The fusion proteins (AhSUCA06‐GFP and AhSUCA16‐GFP) co‐localized with the nuclear marker, indicating that both *AhSUCA06* and *AhSUCA16* are localized to the nucleus (Figure 5b; Figure S8b). These findings suggest that *AhSUCA06* and *AhSUCA16* may be involved in nuclear processes and may function in transcriptional control during peanut seed development.

The potential roles of AhSUCA06 and AhSUCA16 as transcriptional regulators were assessed using a dual‐luciferase reporter (DLR) assay (Figure 5c–e; Figure S8c–e). *AhSUCA06* and *AhSUCA16* expression resulted in a decrease of firefly luciferase/*Renilla* luciferase (LUC/REN) activity compared with the GAL4‐BD negative control (Figure 5d,e; Figure S8d,e). This reduction in luciferase activity indicates that both AhSUCA06 and AhSUCA16 exhibit transcriptional repression activity in this assay system.

Bifc assays demonstrate physical protein–protein interaction between AhSUCA06 and AhSUCA16, which likely function as transcriptional repressors by forming a heterodimeric complex.

### 2.8 Identification of *AhSUCA06* Target Genes

**FIGURE 6:**
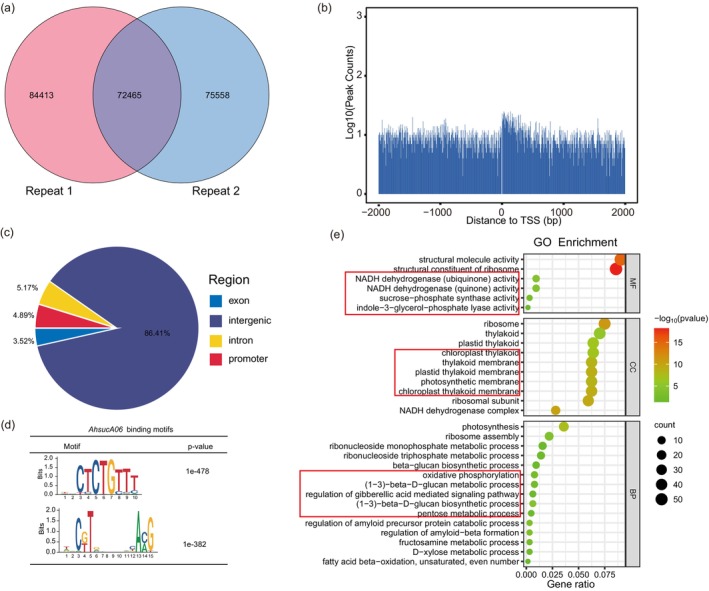
Genome‐wide analysis of AhSUCA06‐binding sites and target genes via DAP‐seq. (a) Overlap of AhSUCA06‐binding peaks in two technical replicates of DAP‐seq. (b) AhSUCA06‐binding sites are enriched in the region proximal to the TSS. (c) Distribution of peaks in different target gene regions. (d) Potential binding motifs for *AhSUCA06* according to DAP‐seq. (e) GO enrichment analysis of *AhSUCA06* target genes.

To identify genome‐wide binding sites of AhSUCA06 and characterize the sequence motifs associated with its binding, DNA affinity purification sequencing (DAP‐seq) was performed (Figure 6a). Binding sites for *AhSUCA06* were distributed evenly within a 2000 bp region upstream and downstream of the transcription start site (TSS) (Figure 6b). Notably, 4.89% of the *AhSUCA06*‐binding sites were located within the promoter region (2 kb upstream of TSS) of 3544 genes, suggesting that *AhSUCA06* may regulate their expression (Figure 6c; Table S13). A MEME (Multiple EM for Motif Elicitation) analysis identified CT(G)C/T and AA(C)G as binding motifs for *AhSUCA06* (Figure 6d).

Because transcription factors (TFs) primarily regulate gene expression by binding to promoter regions, genes with AhSUCA06 binding sites in their promoters were selected for further analyses. Gene Ontology (GO) enrichment analysis showed that these target genes are enriched in GO terms related to ribosome structure, thylakoid membrane function, UDP‐glucose transmembrane transporter activity, sucrose‐phosphate synthase activity, and energy metabolism, including fatty acid beta‐oxidation and nucleoside phosphate metabolism (Figure 6e).

### 2.9 Transcriptomic Validation

Transcriptome sequencing of seeds from T_2_ mutants and wild‐type YH9326 across different developmental stages identified a total of 10 387 differentially expressed genes (DEGs, Figure S9), with each gene showing significant differential expression in at least one stage (|log_2_FC| > 1, *p* < 0.05). KEGG pathway enrichment analysis revealed significant enrichment of these DEGs in multiple metabolic pathways (Figure S10): 769 genes were categorized under carbohydrate metabolism, 370 under amino acid metabolism, and 406 under lipid metabolism. DEGs in these pathways are likely to directly or indirectly regulate the accumulation of sucrose, oil, and protein in seeds. Among the 406 lipid metabolism‐related DEGs, we further identified 79 involved in the fatty acid synthesis pathway.

## 3 Discussion

### 3.1 Impact of Genotype × Environment Interaction on QTL Effects for SSC, SOC and SPC

The significant genotype × environment (G × E) interaction observed for SSC, SOC and SPC (Table 1) underscores the influence of environmental conditions on the expression of these complex traits. Our multi‐environment trials revealed that while the major QTLs *qA06.1* and *qA16.1* were consistently detected across all locations (Yuanyang in 2022 and 2023), explaining a large proportion of PVE, their individual effects showed some variation. For instance, the PVE for *qA16.1* on SOC ranged from 28.0% to 37.4% across environments (Table S8). This variation may be attributed to differences in key environmental factors such as temperature and water availability during the from pod enlargement to seed plumpness stage (Table S15), which are known to influence oil and sugar metabolism pathways. Conversely, the minor QTL *qA08.1* was not consistently detected in all environments, suggesting it may represent a conditional locus whose expression is more sensitive to specific environmental cues. The stability of *qA06.1* and *qA16.1* across diverse conditions enhances their reliability for marker‐assisted selection. More importantly, the functional validation of AhSUCA06 via CRISPR/Cas9 under controlled greenhouse conditions provides direct genetic evidence that transcends G × E interactions, confirming their fundamental regulatory roles in sucrose and oil metabolism. Future breeding efforts should consider testing elite lines carrying favourable alleles of these genes across target environments to fully exploit their potential while managing G × E effects.

### 3.2 Co‐Localization of QTLs for SSC, SOC and SPC

In previous studies, QTLs related to SSC were mapped and the associated molecular mechanisms were examined (Guo et al. 2023; Huai et al. 2024; Li et al. 2021; Wang et al. 2024; Zhao et al. 2025). In a recent study, we mapped a major QTL for sucrose content, *qSUCA06*, in A06:112.36–112.66 Mb (arahy.Tifrunner.gnm2.A16:148.10–148.40 Mb) using an F_2_ population derived from the JHT1 × PI cross (Guo et al. 2023). Two candidate regions related to SSC, *qSUCA08a* (34.66–36.77 Mb) on A08 and *qSUCA16a* (22.60–30.22 Mb) on A16, were identified using an RIL population derived from Zhonghua 10 and ICG 12625 on the basis of QTL‐seq data (Li et al. 2023). Specifically, the candidate region for *qA06.1* (115.43–115.93 Mb) (Figure 2a) partially overlapped a previously reported interval (115.78–115.81 Mb) (Huai et al. 2024). The candidate region for *qA16.1* (148.16–148.56 Mb; Figure 2a) identified in this study showed partial overlap with two recently reported QTL intervals, specifically *qSCB06.2* (147.9–148.6 Mb) and *qSCB06* (148.03–148.16 Mb). The *qSCB06.2* interval was detected in a RIL population using whole‐genome resequencing (Wang et al. 2024), whereas *qSCB06* was mapped in an F_2:3_ population via BSA‐seq (Zhao et al. 2025). Notably, the candidate region on A08 (44.49–46.46 Mb) identified in this study differs from the interval (34.66–36.77 Mb) reported by Li et al. (2023). The genomic locations of the QTLs identified in this study show both consistencies and discrepancies with those reported previously, which can be attributed to several key factors that influence QTL mapping outcomes. Firstly, differences in the genetic backgrounds of mapping populations are a primary source of variation. For instance, while we detected a candidate region on A08 (44.49–46.46 Mb), a distinct interval (34.66–36.77 Mb) was reported by Li et al. (2023), likely because the respective hybrid parents (JHT1 × PI vs. Zhonghua 10 × ICG 12625) carry different allelic variants governing sucrose content. Secondly, variations in mapping resolution and strategy can refine or shift intervals. The partial overlaps observed for other QTLs (e.g., *qA06.1* with Huai et al. 2024; *qA16.1* with Wang et al. 2024 and Zhao et al. 2025) highlight genomic hotspots that are robust across studies, with interval variations often reflecting differences in mapping resolution (e.g., BSA‐seq vs. whole‐genome resequencing).

These observed discrepancies do not diminish the generalizability of our findings but rather contextualize them. They underscore that seed sucrose content is regulated by a complex genetic architecture, with both conserved and population‐specific QTLs. The core value of our study lies not merely in the QTL coordinates, but in the subsequent functional validation of the candidate genes *AhSUCA06* and *AhSUCA16* within the identified intervals. This validation step transcends population‐specificity and provides direct mechanistic insight, thereby offering universally applicable targets for marker‐assisted selection aimed at manipulating sucrose metabolism in peanut breeding.

The co‐localization of QTLs for SSC, SOC and SPC revealed in this study (Table S8) is in accordance with the findings of an earlier study, which detected co‐localized QTLs for SSC and SOC (Huai et al. 2024). In soybean, QTLs controlling 100‐seed weight, protein content and oil content were co‐localized on chromosome 15 (Yang et al. 2019). Moreover, *GmSWEET10a* expression reportedly leads to increases in soybean seed size and the oil content, but decreases in the protein content (Wang et al. 2020). Sweet corn results from mutations in genes related to starch synthesis pathways; these mutations alter carbohydrate compositions by increasing the sugar content and decreasing the starch content in the endosperm (Revilla et al. 2021). A recent study showed that in corn, the expression of *ZmCCD8*, which is a key strigolactone synthase gene, simultaneously affects the regulation of sucrose and amino acid contents and grain size (Zhong et al. 2025). In Arabidopsis and soybean wrinkled leaf mutants, a significant decrease in the oil content is accompanied by an increase in the sucrose content (Cernac and Benning 2004). Relationships among SSC, SPC and SOC suggest that these traits may be regulated by a complex molecular network.

### 3.3 Epistasis Between *qA06.1* and *qA16.1*

A recent study identified two major QTLs (*qSCA06.2* and *qSCA16*.*2*) using a high‐density genetic map constructed on the basis of a whole‐genome resequencing analysis of a peanut RIL population (Wang et al. 2024). These two QTLs were found to have synergistic regulatory effects on the sucrose content (Wang et al. 2024). In the current study, *qA06*.*1* and *qA16.1* were found to synergistically modulate SSC, SOC and SPC (Figure 3a; Figure S7). In two RIL populations (Figure S7), accessions in which both genes were mutated had high SSC and SPC, but low SOC. Interestingly, there were no significant differences in SSC, SPC and SOC between accessions with only one mutation (i.e., aaBB and AAbb) and the WT control (i.e., AABB). An analysis of epistasis confirmed the interactions among the *qA06*.*1* and *qA16.1* synergistically regulate SSC, SOC and SPC. This gene dosage effect is analogous to the regulation of the oleic acid content by *FAD2* in peanut (Barkley et al. 2013; Nawade et al. 2016; Yu et al. 2008). Additionally, similar significant phenotypic changes due to simultaneous mutations in two copies of a single gene have been observed in grape (Walker et al. 2007), apple (Costa et al. 2010) and wheat (Su et al. 2011).

### 3.4 *AhSUCA06* and *AhSUCA16* Encode Negative Regulators of SSC

In this study, *AhSUCA06* and *AhSUCA16* were identified as homologues that co‐regulate the sucrose content in peanut seeds. In JHT1, a C‐to‐A substitution was detected in the *AhSUCA06* CDS, which resulted in premature translation termination. Subcellular localization results indicated that *AhSUCA06* is localized in the nucleus, which is consistent with the findings of a recent study (Huai et al. 2024). In addition, a comparison of *AhSUCA16* sequences in PI and JHT1 revealed a fragment deletion in *AhSUCA16* in JHT1. Subcellular localization results indicated that *AhSUCA16* is localized in the nucleus. Furthermore, according to the results of a DLR assay, *AhSUCA06* and *AhSUCA16* may encode proteins that repress transcription, suggesting that they may be regulators of target gene expression. CRISPR/Cas9‐mediated genetic transformation experiments further demonstrated that knocking out *AhSUCA06* can significantly increase SSC and SPC, but decrease SOC. The function of the AhSUCA06 protein has not been reported. According to the reference genome annotation, this gene encodes an uncharacterized protein, as also reported by Huai (Huai et al. 2024). Analysis via the InterPro (https://www.ebi.ac.uk/interpro/search/sequence/) and UniProt (https://www.uniprot.org/blast) databases predicts that the protein contains a DUF7950 domain. Studies on this domain in Arabidopsis, wheat, and maize suggest its potential association with seed dormancy and germination processes. However, comparative analyses using the NCBI (https://www.ncbi.nlm.nih.gov/Structure/cdd/wrpsb.cgi) and the KEGG (http://www.genome.jp) still annotate this protein as functionally unknown; a PROSITE database scan also failed to identify any known functional motifs. In summary, the specific biochemical function of *AhSUCA06* remains to be elucidated.

To decipher its function, subsequent research plans include: resolving its three‐dimensional structure through cryo‐electron microscopy combined with AI modelling; screening for its interacting proteins during seed development using yeast two‐hybrid or Co‐IP‐MS techniques; and conducting enzyme activity assays following the in vitro expression and purification of this protein to reveal its potential biochemical activities. CRISPR/Cas9‐mediated genetic transformation experiments confirmed that *AhSUCA06* and *AhSUCA16* collaboratively regulate SSC, SOC and SPC. Analysis of DAP‐seq data suggests that their phenotypic effects may arise from the modulation of gene expression in pathways related to glycolysis, gluconeogenesis and lipid metabolism. However, this molecular mechanism still requires further experimental validation.

### 3.5 Conclusion

In this study, *qA06.1* and *qA16*.*1* were identified as genomic regions associated with SSC, SOC and SPC on the basis of BSA‐seq, linkage mapping, and epistasis analyses. CRISPR/Cas9‐mediated genetic transformation experiments indicated that the knockout of *AhSUCA06* and *AhSUCA16* increases SSC and SPC in peanut kernels, while decreasing SOC. Subcellular localization and DLR assays revealed that *AhSUCA06* and *AhSUCA16* are nuclear‐localized proteins and possess transcriptional repressor activity, implying that they are involved in regulating gene expression. Further research is necessary to further elucidate *AhSUCA06* and *AhSUCA16* interacting partners, as well as the mechanisms by which these proteins regulate SSC, SOC and SPC.

## 4 Experimental Procedures

### 4.1 Plant Materials and Field Trials

Two RIL populations were derived from reciprocal crosses between JHT1 and PI in 2015; the JHT1 × PI RIL population (JP‐RIL) and PI × JHT1 RIL population (PJ‐RIL) consisted of 422 and 312 F_9:11_ lines, respectively. JHT1, which is a peanut variety with high SSC, high SPC and low SOC, was released by Hebei Academy of Agriculture and Forestry Sciences (China), whereas PI, which has low SSC, low SPC and high SOC, is a germplasm from the United States. The two RIL populations and two parents were grown in Yuanyang (113°55′ E, 35°18′ N), China in 2022 (F_8_ generation; E1) and 2023 (F_9_ generation; E2). Additionally, a natural peanut panel comprising 353 cultivated tetraploid accessions genotyped by whole‐genome resequencing was used to verify the genetic effects of major QTLs (Zheng et al. 2024). They were grown in Zhengzhou (113°41′ E, 34°45′ N; E3), Shangqiu (116°39′ E, 34°52′ N; E4) and Weifang (119°09′ E, 36°42′ N; E5), China in 2019. YH9326, which is a peanut variety (with SSC of ~4.5%), was bred by the Henan Academy of Agricultural Sciences (China) and serves as a transgenic recipient for peanuts. Transgenic lines and YH9326 were cultivated in a greenhouse with a 16‐h light (28°C)/8‐h dark (25°C) cycle.

Field experiments were conducted using a randomized complete block design with two replicates. Ten seeds were sown in a plot with rows that were 1.8 m long and separated by 0.4 m.

### 4.2 Trait Measurements

For each line in the RIL population (E1 and E2) and germplasm collection (E4 and E5), approximately 30 mature, plump, and disease‐ and pest‐free peanut seeds were selected for an analysis of SSC, SOC and SPC via near‐infrared reflectance spectroscopy using the Grain Quality Analyser DA7200 (Perten Instruments (Beijing) Co. Ltd., China) (Guo et al. 2023). In addition, SSC was measured for the natural peanut population in E3 and SPC was determined for transgenic lines using the same methods.

SOC was measured for transgenic lines and the natural peanut panel in E3 according to a Soxhlet extraction method (Huai et al. 2024). SSC was measured for transgenic lines using a high‐performance liquid chromatography–charged aerosol detection system (Huai et al. 2024). SPC was measured for the natural peanut panel in E3 according to a Kjeldahl method (ISO 20483:2013) using an automated analyser (FOSS Kjeltec 8400, Denmark).

### 4.3 Genomic DNA, Total RNA Isolation and Quantitative Real‐Time PCR (qRT‐PCR)

Genomic DNA (gDNA) was extracted from young leaves using the Plant Genome DNA Extraction Kit (DP305‐03, Tiangen). Total RNA for all the samples was extracted from JHT1 and PI using an RNA Extraction Plant kit (Takara, Otsu, Japan). Purified total RNA (1 μg) was reverse transcribed by priming with oligo dT (15) in a 20 μL reaction volume using a Reverse Transcriptase kit (A3500, Promega, USA). The qRT‐PCR was performed using Power UpTM SYBRTM Green ‐Master Mix (Applied Biology Inc., Irvine, CA, United States) on a real‐time PCR detection system according to the manufacturer's instruction (ABI, USA). The relative expression levels of each gene among different samples were calculated using the 2^−ΔCt^ method and normalized by the internal reference *ADH3* gene (Brand and Hovav 2010; Livak and Schmittgen 2001). Three biological replicates of each sample type were collected and three technical replicates were performed for each biological replicate. Primers used for qRT‐PCR are listed in Table S14.

### 4.4 Bulked Pool Construction and Whole‐Genome Resequencing

On the basis of SSC of JP‐RIL in E2, 51 lines with high SSC, high SPC and low SOC and 41 RILs with low SSC, low SPC and high SOC were selected to construct HSP and LSP, respectively. Equal amounts of DNA from each line in HSP and LSP were pooled. The two bulked pools and the parental genomic DNA were used for the WGS, which was completed using the Illumina HiSeq 2000/MiSeq platform to generate 150‐bp paired‐end reads. Sequencing depths for the bulked pools and two parents were 30× and 20×, respectively.

### 4.5 BSA‐Seq Analysis

After adapters and low‐quality reads were eliminated, the remaining clean reads were aligned to the reference genome ( *A. hypogaea* cv. Tifrunner v2.0) (Bertioli et al. 2019) using BWA (version 0.7.10) (Li and Durbin 2009). On the basis of uniquely mapped reads, SNPs were called using SAMtools (version 0.1.19) and GATK (version 3.3.0) (McKenna et al. 2010). Homozygous and polymorphic SNPs between the parents were retained for BSA. Additionally, Δ(SNP‐index), Euclidean distance (ED), G‐statistic, and *p* values were calculated using a 2‐Mb sliding window with a step size of 10 kb. A 99% confidence level was selected as the threshold for detecting candidate regions for SSC.

### 4.6 KASP Marker Development, Genetic Map Construction and QTL Mapping

Polymorphic SNPs (between the two parents) in the candidate region were converted to KASP markers (Trick et al. 2012) and then the lines of both RIL populations were genotyped. Genetic linkage maps were constructed using Joinmap (version 5.0) (van Ooijen 2018) on the basis of LOD scores ranging from 2 to 28. The maximum likelihood algorithm was used to determine marker order, with Kosambi's mapping function applied to calculate genetic distance. The genetic map was plotted using the R package LinkageMapView (Ouellette et al. 2018). A QTL analysis was performed using multiple QTL mapping (MQM) of MapQTL (version 6.0) (Van Ooijen 2009), with a mapping step size of 0.1 cM and an LOD threshold of 2.5. An epistatic QTL (Epi‐QTL) was detected for SSC, SOC and SPC using the ICIM‐EPI module of QTL IciMapping (version 4.2) (Meng et al. 2015).

### 4.7 Candidate Gene Prediction and Sequence Analysis

The reference genome ( *A. hypogaea* cv. Tifrunner v2.0) (Bertioli et al. 2019) and SnpEff 4.2 software (Cingolani et al. 2012) were used to locate genomic variants and predict their effects on known genes at loci containing QTLs. On the basis of the reference genome, candidate gene functions were annotated.

Candidate genes were amplified by PCR from developing PI and JHT1 seeds at 55 days after flowering. Multiple sequence alignments were performed using Clustal W (Thompson et al. 1994). Primers used for PCR amplification are listed in Table S14.

### 4.8 Functional Verification of *AhSUCA06* and *AhSUCA06*

Vectors used to functionally characterize *AhSUCA06* and *AhSUCA06* were constructed as previously described (Xue et al. 2025). Because the *AhSUCA16* sequence in JHT1 is identical to that in peanut cultivar YH9326, a CRISPR/Cas9‐BGK012 vector (Wimi Biotechnology, Nanjing, China) was constructed using an AtU6‐driven sgRNA targeting the *AhSUCA06* CDS. The vector was then inserted into YH9326 via particle bombardment (Xue et al. 2025). Bombarded embryogenic calli were screened on selection medium containing 10–20 mg/L hygromycin B, with putative transformants transferred to induction, shoot proliferation, and plantlet development media to regenerate whole seedlings. Positive and negative CRISPR/Cas9 T_0_ lines were grafted onto YH9326 rootstock (3–5 plants per line). Transgenic lines were analysed by sequencing using a high‐throughput mutation detection platform (Liu et al. 2019). Primers used for PCR amplification are listed in Table S14.

### 4.9 Subcellular Localization Assay

*AhSUCA06* and *AhSUCA16* CDSs without the termination codon were cloned into separate pBWA(V)HS‐GFP vectors (provided by BioRun Biotechnology Co. Ltd., Wuhan, China) to generate the fusion constructs pBW‐AhSUCA06‐GFP and pBW‐AhSUCA16‐GFP, respectively. Recombinant vectors were inserted into *Agrobacterium tumefaciens* strain GV3101 cells, which were then infiltrated into *Nicotiana benthamiana* leaves. The GFP signal was examined at 48 h post‐infiltration using a confocal laser‐scanning microscope (Olympus FluoView FV1000, Olympus, Tokyo, Japan). NLS‐mKate, a nuclear marker consisting of the far‐red fluorescent protein mKate with an N‐terminal nuclear localization sequence (NLS), was used as a reference (Ji et al. 2019; Liu et al. 2018). Primers used for PCR amplification are listed in Table S14.

### 4.10 Bimolecular Fluorescence Complementation (BiFC) Assays

The coding sequences of *AhSUCA06* and *AhSUCA16* were cloned into the pSPYNE and pSPYCE vectors, respectively. Co‐expression analysis was conducted in epidermal cells of *Nicotiana benthamiana* leaves as described in previous studies (e.g., Li et al. 2018). Negative controls included the combination of AhSUCA16‐pSPYCE with empty pSPYNE and AhSUCA06‐pSPYNE with empty pSPYCE. Reconstituted YFP fluorescence was examined using a laser scanning confocal microscope to assess protein–protein interactions in planta.

### 4.11 Transcriptional Activity and Transient Expression Regulation Assays

A DLR assay was performed as previously described with minor modifications (Hellens et al. 2005). Two reporter constructs were used. The first, containing the LUC gene fused to the 5× GAL4 upstream activation sequence, was inserted into the pGreenII 0800‐LUC vector with a downstream mini35S promoter. The other reporter construct included the REN gene as an internal control for normalization. For the effector constructs, the CDSs of AhSUCA06 and AhSUCA16 were fused in frame to the GAL4 DNA‐binding domain (BD) in pGreenII62‐SK‐GAL4, allowing the fusion proteins to bind GAL4‐responsive elements in the reporter construct. The VP16 activation domain was fused in frame to the GAL4 DNA‐binding domain in pGreenII62‐SK‐GAL4 to generate the positive control (BD–VP16), whereas the empty GAL4‐BD vector (BD) served as the negative control.

The reporter and effector vectors were inserted into *A. tumefaciens* strain GV3101 cells for the co‐transformation of young *N. benthamiana* leaves. After 48 h post‐transformation, firefly and *Renilla* luciferase signals were detected using a Dual‐Luciferase Assay Kit (DL101‐01, Vazyme Biotech, Nanjing, China). Briefly, leaves were immersed in D‐luciferin sodium salt solution (Vazyme Biotech, Nanjing, China) and then luminescent signals were captured using an SH‐Compact 523 chemiluminescence imaging system (Shenhua, Hangzhou, China).

### 4.12 DAP‐Seq Analysis

A DAP‐seq assay was performed as previously described (Bartlett et al. 2017). A gDNA library was constructed using PI peanut kernels at 55 days after flowering. Briefly, the *AhSUCA06* CDS was cloned into the pFN19K HaloTag T7 SP6 Flexi expression vector (provided by BioRun Biotechnology Co. Ltd., Wuhan, China). Protein‐bound beads were incubated with gDNA fragments, after which eluted DNA fragments were sequenced using an Illumina NovaSeq 6000 platform. Clean reads were mapped to the reference genome using BWA (version 0.7.17‐r1188) (Li and Durbin 2010). In addition, MACS2 software was used to call peaks and calculate peak scores (Zhang et al. 2008). Two independent replicates were merged and annotated using bedtools (version 2.30.0) (Quinlan and Hall 2010), with peaks within 2000 bp from the TSS considered to be localized in the promoter region. Binding motifs within peak regions were analysed using HOMER (Hypergeometric Optimization of Motif EnRichment) (version 4.11) (http://homer.ucsd.edu/homer/motif/index.html), whereas the frequency distribution of peaks near TSS was analysed using deepTools (Ramírez et al. 2016). *AhSUCA06* binding peaks were visualized using Integrative Genomics Viewer. Additionally, a GO analysis was completed using an online program (https://www.bioinformatics.com.cn/) to further examine DNA features and their associated functions.

### 4.13 Transcriptome Sequencing Analysis

Seed samples from the wild‐type (YH9326) and the homozygous mutant (C#30‐1, KO30‐1) were collected at 35, 55, 75 and 85 days after flowering (DAF), with three biological replicates for each sample. RNA sequencing was performed by using the Illumina NovaSeq 6000 platform. After filtering raw reads, clean data were aligned to the peanut *A. hypogaea* cv. Tifrunner v2.0 reference genome. Four comparison groups were designed: KO30‐1_35DAF versus YH9326_35DAF, KO30‐1_55DAF versus YH9326_55DAF, KO30‐1_75DAF versus YH9326_75DAF and KO30‐1_85DAF versus YH9326_85DAF. Differentially expressed genes (DEGs) were identified using edgeR with the thresholds |log_2_FC| ≥ 1 and *p* ≤ 0.05. Volcano plots of DEGs were generated using OmicStudio (https://www.omicstudio.cn/home). Functional enrichment analyses, including KEGG and GO, were conducted. Venn diagrams of DEGs were plotted using the online tool Eveen.

### 4.14 Statistical Analysis of Phenotyping Data

Basic statistical analyses were conducted using. The descriptive statistics analysis, correlation analysis, one‐way analysis of viarance, Student's *t*‐test and multiple comparisons test were performed using the SPSS (version 20.0) (IBM SPSS, Chicago, IL). The combined analysis of variance (ANOVA) for SSC, SOC and SPC was conducted using the AOV module in QTL IciMapping (version 4.2) (Meng et al. 2015). A linear model was fitted with genotype and environment as fixed effects, including their interaction term. The significance of the genotype, environment, and genotype × environment interaction effects was evaluated with the F‐test, using a significance threshold of *p* < 0.05. Broad‐sense heritability (*H* ^2^) was estimated from the variance components derived from this model. Best linear unbiased prediction (BLUP) values for SSC, SOC and SPC of JP‐RIL and PJ‐RIL in two environments were calculated using the lme4 package in R (Bates et al. 2015). Frequency distributions as well as the results of correlation analyses and multiple comparison tests for SSC, SOC and SPC were visualized using Origin 2022. Histograms and boxplots were created using GraphPad Prism 9.5.

## Author Contributions

X.Z. and Z.Z. conceived and designed the study. Y.Z., F.Q. and Z.S. prepared DNA and RNA samples and performed laboratory experiments. H.L., L.Q., J.W., C.Z., M.W., Z.M., X.W., H.L., Y.H. and W.D. assisted in laboratory and field experiments, and L.S., H.Z., X.L. provided services in peanut gene editing experiments. Y.Z. and F.Q. performed bioinformatics analyses and wrote the manuscript. X.Z. and S.P. revised the manuscript. All authors read and approved the final manuscript.

## Conflicts of Interest

The authors declare no conflicts of interest.

## Supporting information


**Figure S1:** Phenotypic distributions of the seed sucrose content (SSC), seed oil content (SOC) and seed protein content (SPC) in RIL populations across two environments. (a) Frequency distributions of SSC, SOC and SPC in the JP‐RIL population. (b) Frequency distributions of SSC, SOC and SPC in the PJ‐RIL population.

**Figure S2:** QTLs for SSC, SOC and SPC identified on the basis of BSA‐seq. (a) Manhattan plot showing the distribution of Δ(SNP‐index). (b) Distribution of −log_10_(*P* values) derived from Fisher's exact tests. Green/blue and red lines represent 95% and 99% confidence intervals, respectively. Black lines indicate the average value for four algorithms according to a sliding window analysis. Red boxes indicate significant regions for SSC, SOC and SPC.

**Figure S3:** QTLs and LOD curves for SSC, SOC and SPC in the PJ‐RIL population on LG06 (a), LG08 (b) and LG16 (c), with red boxes indicating candidate regions for SSC, SOC and SPC.

**Figure S4:** Alignment of *AhSUCA06* sequences amplified by PCR. (a) Alignment of *AhSUCA06* sequences in JHT1 (Mu) and PI (WT); *arahy.42CAD1* is the reference genome sequence. (b) Alignment of protein sequences encoded by *AhSUCA06* in JHT1 (Mu) and PI (WT). The mutation site is indicated by a red box.

**Figure S5:** Alignment of *AhSUCA16* sequences in JHT1 (Mu) and PI (WT) by PCR; *arahy.3URM83* is the reference genome sequence. The InDel locus is indicated by a red box.

**Figure S6:** Alignment of protein sequences encoded by *AhSUCA16* in JHT1 (Mu) and PI (WT); *arahy.3URM83* is the reference genome sequence. The InDel locus is indicated by a red box.

**Figure S7:** Validation of the genetic effects of KASP markers Tif2.A06.115805462 and Tif2.A16.148167815 associated with a QTL for SSC, SOC and SPC in two RIL populations in two environments. (a) Genotypic diversity of Tif2.A06.115805462 and Tif2.A16.148167815 in the JP‐RIL population across two environments. (b) Genotypic diversity of Tif2.A06.115805462 and Tif2.A16.148167815 in the PJ‐RIL population across two environments. Asterisks indicate significant differences (one‐way ANOVA; *p < 0.05, **p < 0.01, ***p < 0.001).

**Figure S8:** Molecular characterization of *AhSUCA16*. (a) *AhSUCA16* expression levels in various organs revealed by qRT‐PCR. R, root; S, stem; L, leaf; F, flower; P, peg; SK: seed kernels. Data are presented as the mean ± standard error of three biological replicates. Asterisks indicate significant differences between SK and other organs (Student's *t*‐test; ***p < 0.001). (b) Subcellular localization of *AhSUCA16*. AhSUCA16‐GFP fusion protein was co‐localized with the nuclear marker in tobacco leaf epidermal cells. Scale bars, 50 μm. (c–e) Transcriptional activity analysis of *AhSUCA16* using the DLR assay system. (c) Schematic representation of reporter and effectors. (d) Representative image of a tobacco leaf at 48 h after infiltration. (e) Measurement of relative luciferase activity (LUC/REN). Data are presented as the mean ± standard error of three biological replicates. Asterisks indicate significant differences between BD‐AhSUCA16 and the control group (Student's *t*‐test; ***p < 0.001).

**Figure S9:** Detection of the interaction of interacted AhSUCA06 with AhSUCA16 using bimolecular fluorescence complementation assay. The CDS of *AhSUCA06* and *AhSUCA16* were ligated into the pSPYNE (nYFP) and pSPYCE (cYFP) vectors, respectively, to generate AhSUCA16‐cYFP and AhSUCA06 nYFP vectors. AhSUCA16‐pSPYCE + cYFP and AhSUCA06‐pSPYNE + nYFP were used as negative controls. These vectors were co‐transformed transiently into *Nicotiana benthamiana* mediated by *Agrobacterium tumefaciens* . Bar = 50 μm.

**Figure S10:** Transcriptome analysis was performed using seeds of KO30‐1 and YH9326 collected at 35, 55, 75 and 85 days after flowering (DAF). (a) The number of DEGs between KO30‐1 and YH9326 at different seed developmental stage. (b) Venn diagram analysis of up‐ and down‐regulated differentially expressed genes. (c) Distribution of differentially expressed genes across KEGG pathway categories.

**Table S1:** Descriptive statistics and genetic heritability (H^2^) for seed sucrose content (SSC), seed oil content (SOC) and seed protein content (SPC) in the two parents and PI×JT1 RIL population (PJ‐RIL).
**Table S2:** Correlation analysis of the SSC, SOC and SPC in JP‐RIL by BLUP.
**Table S3:** Correlation analysis of the SSC, SOC and SPC in PJ‐RIL by BLUP.
**Table S4:** SSC, SOC and SPC of constructed extreme mixed pool lines.
**Table S5:** The summary of the sequencing quality and alignment results for BSA‐seq analysis.
**Table S6:** QTLs associated with SSC, SOC and SPC identified using BSA‐seq based on ΔSNP‐index, G‐statistic (G), Fisher‐exact test (Fet) and Euclidean distance (ED).
**Table S7:** KASP primer pairs used for linkage maps construction.
**Table S8:** Summary of significant QTL for the SSC, SOC and SOC in two RIL populations.
**Table S9:** Detail description of epistatic QTLs for the SSC, SOC and SPC in two RIL populations.
**Table S10:** Analysis of mutation sites in candidate genes within the OTLs interval.
**Table S11:** The analysis of mutation types arising from CRISPR/Cas9‐induced callus of T_0_.
**Table S12:** Analysis mutation type in transgenic knockout lines for grafting.
**Table S13:** The target genes in the promoter region identified as *AhSUCA06* binding sites by DAP‐seq analysis.
**Table S14:** Sequences of primers used in this study.

## Data Availability

The data that supports the findings of this study are available in the Supporting Information of this article.
