# Supplementary figures and images for: Mapping and Functional Characterization of Homologous Genes AhSUCA06 and AhSUCA16 Underlying Sucrose, Oil and Protein Contents in Peanut ( Arachis hypogaea L.)
Source: Plant Biotechnol J. 2026 Apr 18;24(8):4844–59. doi: 10.1111/pbi.70667 (PMC13387884; doi:10.1111/pbi.70667)

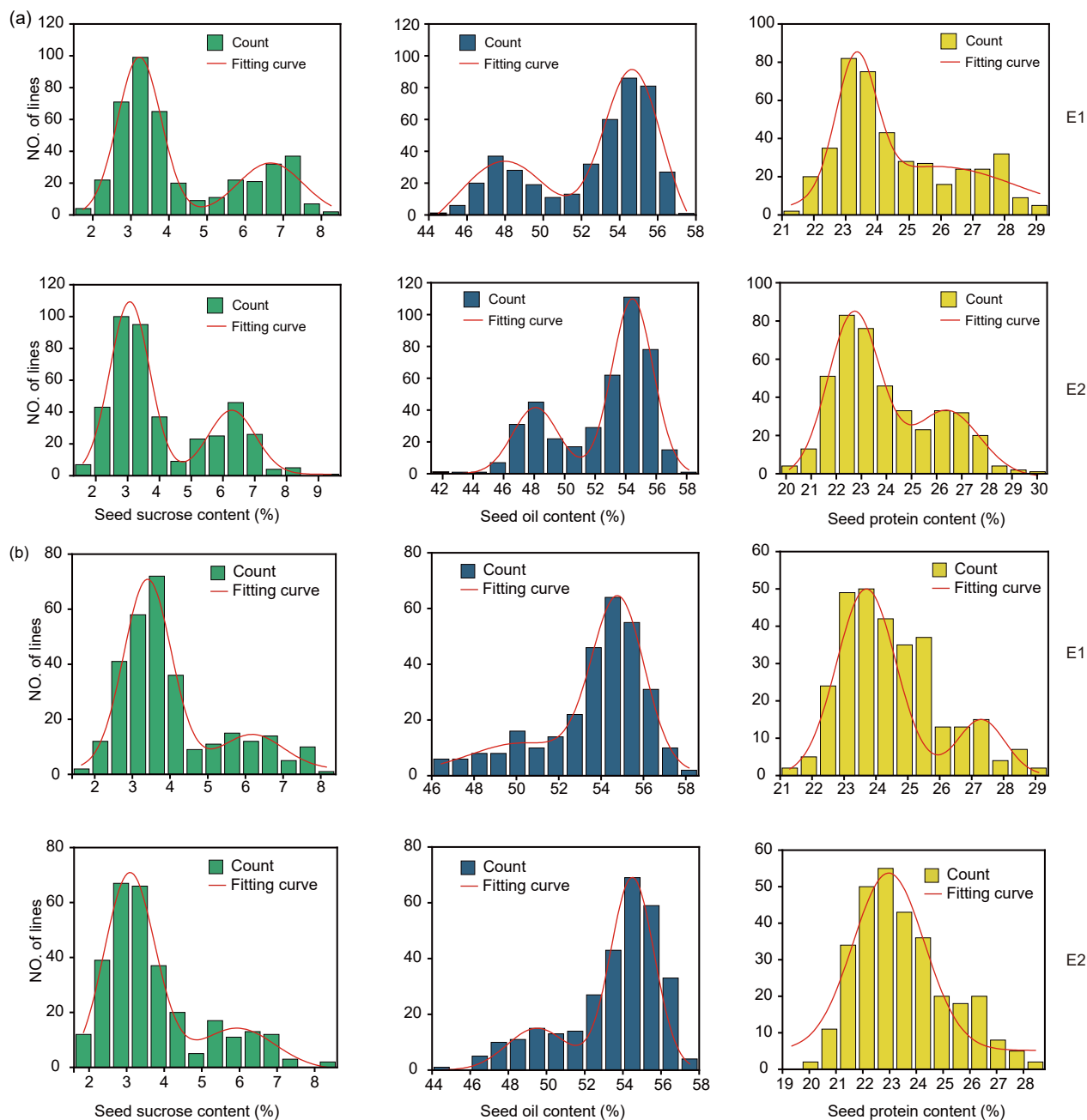

Supplement: Supplementary file 1 — Figure S1: Phenotypic distributions of the seed sucrose content (SSC), seed oil content (SOC) and seed protein content (SPC) in RIL populations across two environments. (a) Frequency distributions of SSC, SOC and SPC in the JP‐RIL population. (b) Frequency distributions of SSC, SOC and SPC in the PJ‐RIL population. [file PBI-24-4844-s001.pdf]

(a)

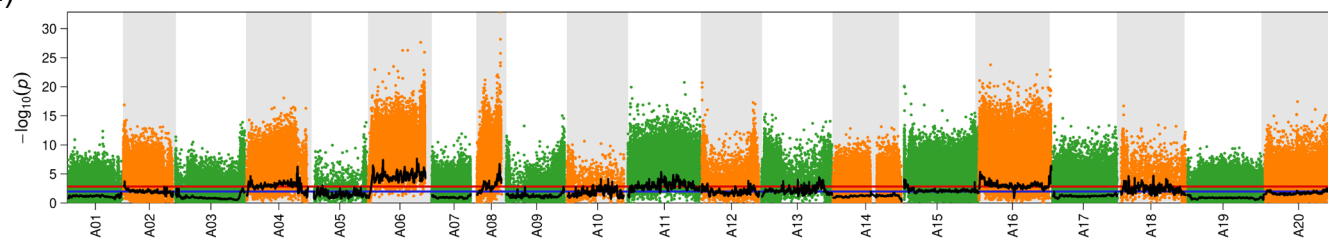

(b)

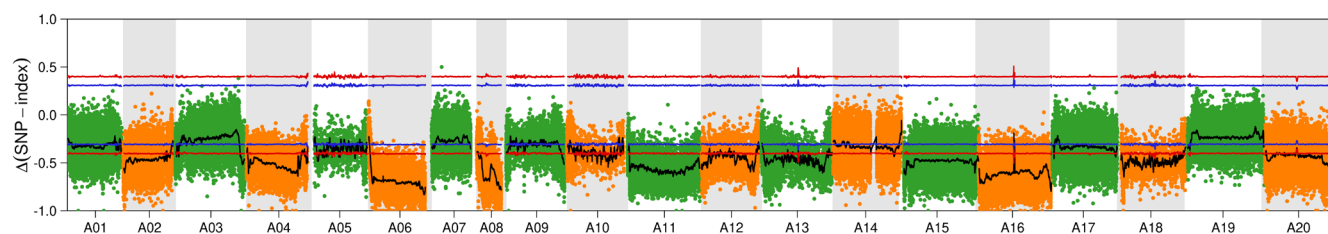

Supplement: Supplementary file 2 — Figure S2: QTLs for SSC, SOC and SPC identified on the basis of BSA‐seq. (a) Manhattan plot showing the distribution of Δ(SNP‐index). (b) Distribution of −log10(P values) derived from Fisher's exact tests. Green/blue and red lines represent 95% and 99% confidence intervals, respectively. Black lines indicate the average value for four algorithms according to a sliding window analysis. Red boxes indicate significant regions for SSC, SOC and SPC. [file PBI-24-4844-s010.pdf]

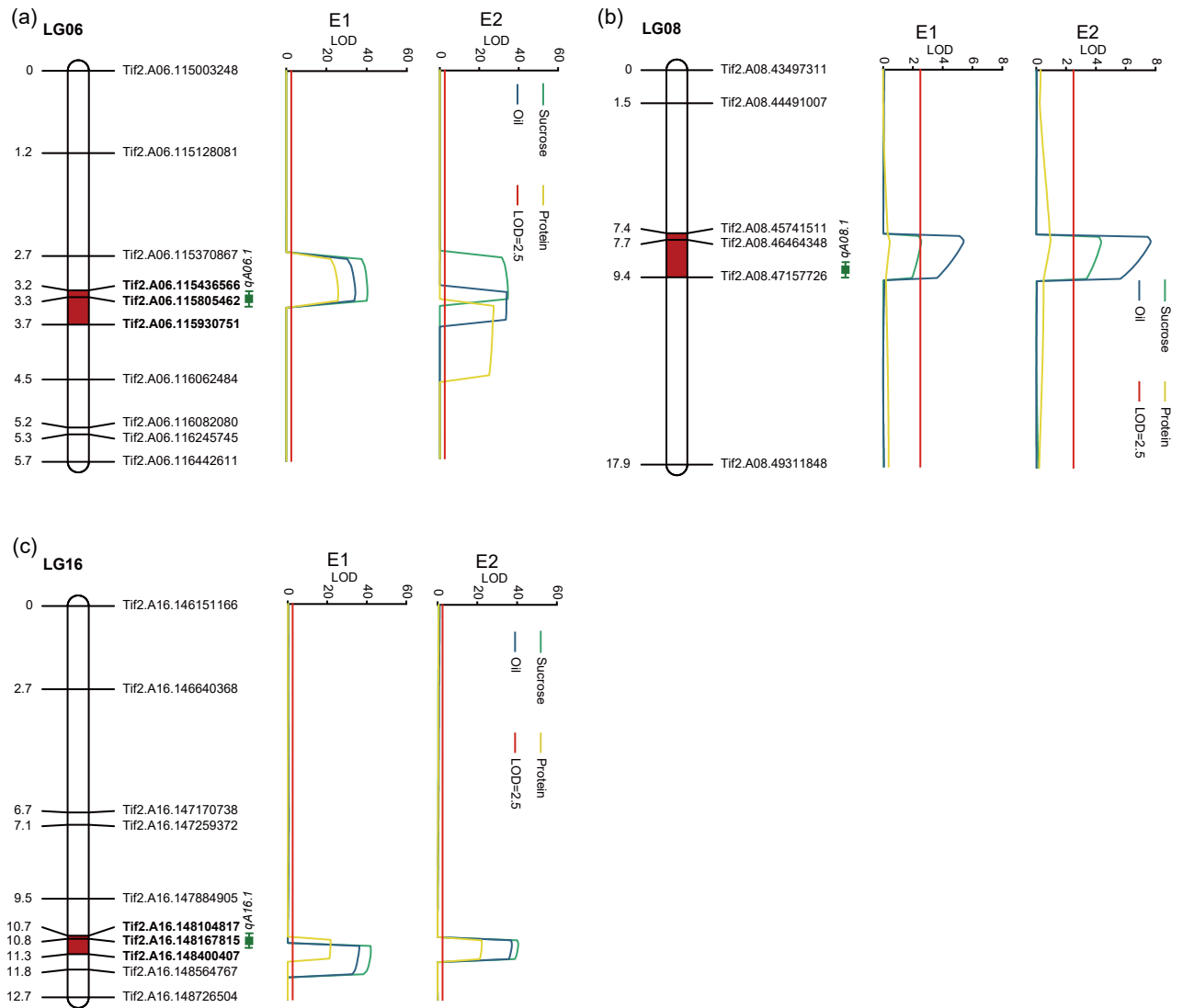

Supplement: Supplementary file 3 — Figure S3: QTLs and LOD curves for SSC, SOC and SPC in the PJ‐RIL population on LG06 (a), LG08 (b) and LG16 (c), with red boxes indicating candidate regions for SSC, SOC and SPC. [file PBI-24-4844-s008.pdf]

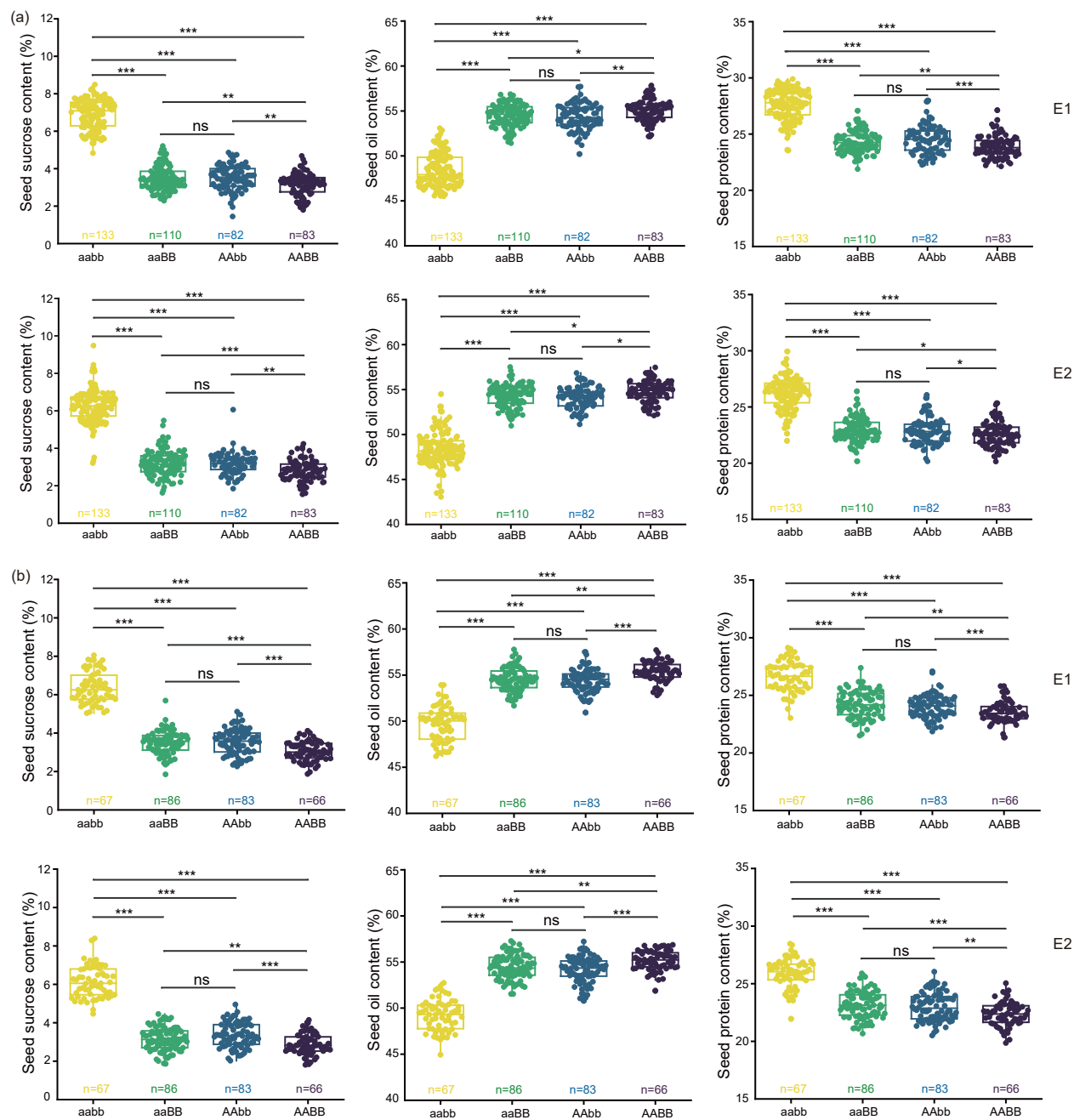

Supplement: Supplementary file 7 — Figure S7: Validation of the genetic effects of KASP markers Tif2.A06.115805462 and Tif2.A16.148167815 associated with a QTL for SSC, SOC and SPC in two RIL populations in two environments. (a) Genotypic diversity of Tif2.A06.115805462 and Tif2.A16.148167815 in the JP‐RIL population across two environments. (b) Genotypic diversity of Tif2.A06.115805462 and Tif2.A16.148167815 in the PJ‐RIL population across two environments. Asterisks indicate significant differences (one‐way ANOVA; *p < 0.05, **p < 0.01, ***p < 0.001). [file PBI-24-4844-s007.pdf]

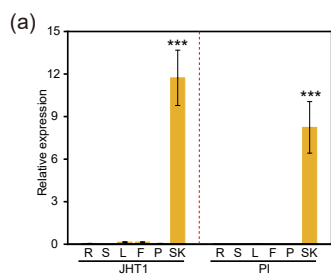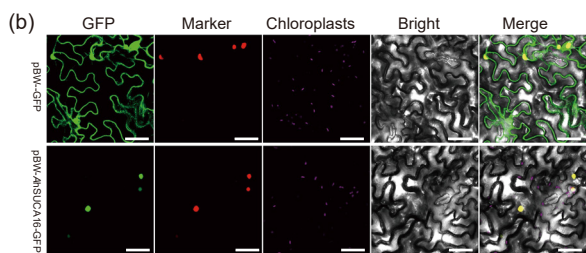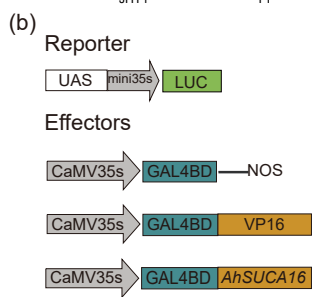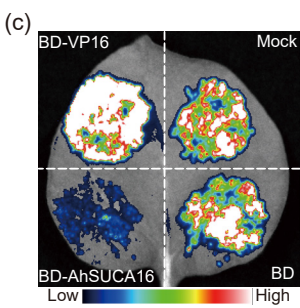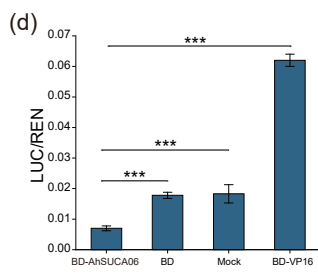

Supplement: Supplementary file 8 — Figure S8: Molecular characterization of AhSUCA16. (a) AhSUCA16 expression levels in various organs revealed by qRT‐PCR. R, root; S, stem; L, leaf; F, flower; P, peg; SK: seed kernels. Data are presented as the mean ± standard error of three biological replicates. Asterisks indicate significant differences between SK and other organs (Student's t‐test; ***p < 0.001). (b) Subcellular localization of AhSUCA16. AhSUCA16‐GFP fusion protein was co‐localized with the nuclear marker in tobacco leaf epidermal cells. Scale bars, 50 μm. (c–e) Transcriptional activity analysis of AhSUCA16 using the DLR assay system. (c) Schematic representation of reporter and effectors. (d) Representative image of a tobacco leaf at 48 h after infiltration. (e) Measurement of relative luciferase activity (LUC/REN). Data are presented as the mean ± standard error of three biological replicates. Asterisks indicate significant differences between BD‐AhSUCA16 and the control group (Student's t‐test; ***p < 0.001). [file PBI-24-4844-s003.pdf]

YFP

Bright

Merge

AhSUCA16-cYFP  
+nYFP

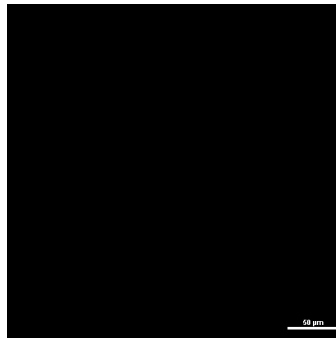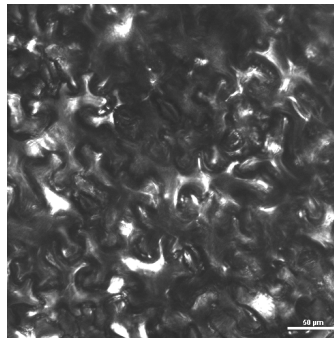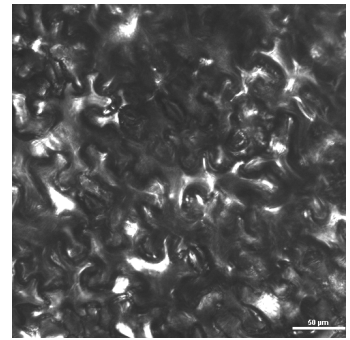

cYFP  
+AhSUCA06-nYFP

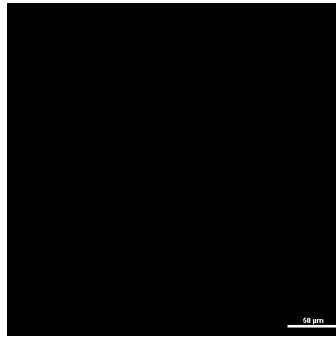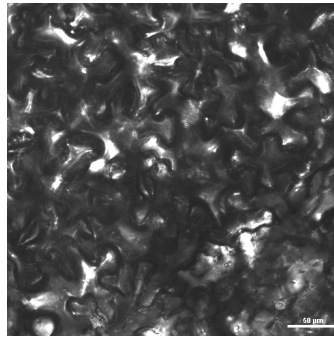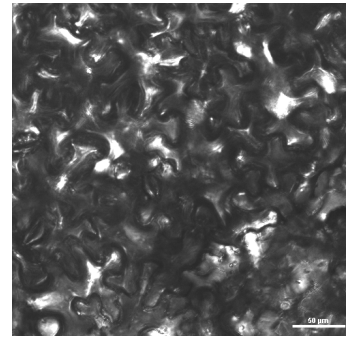

AhSUCA16-cYFP  
+AhSUCA06-nYFP

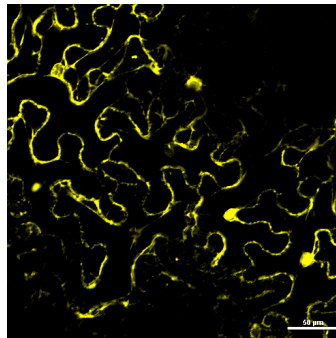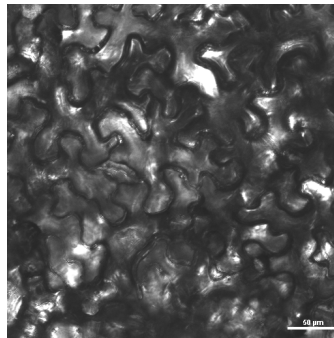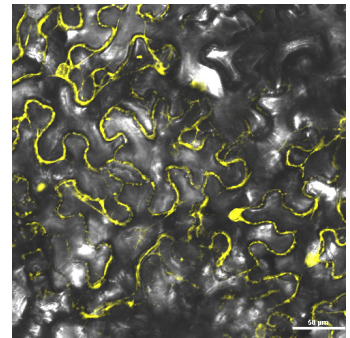

Supplement: Supplementary file 9 — Figure S9: Detection of the interaction of interacted AhSUCA06 with AhSUCA16 using bimolecular fluorescence complementation assay. The CDS of AhSUCA06 and AhSUCA16 were ligated into the pSPYNE (nYFP) and pSPYCE (cYFP) vectors, respectively, to generate AhSUCA16‐cYFP and AhSUCA06 nYFP vectors. AhSUCA16‐pSPYCE + cYFP and AhSUCA06‐pSPYNE + nYFP were used as negative controls. These vectors were co‐transformed transiently into Nicotiana benthamiana mediated by Agrobacterium tumefaciens . Bar = 50 μm. [file PBI-24-4844-s011.pdf]

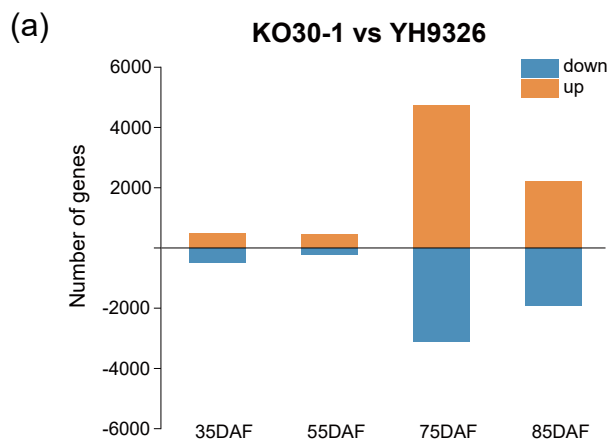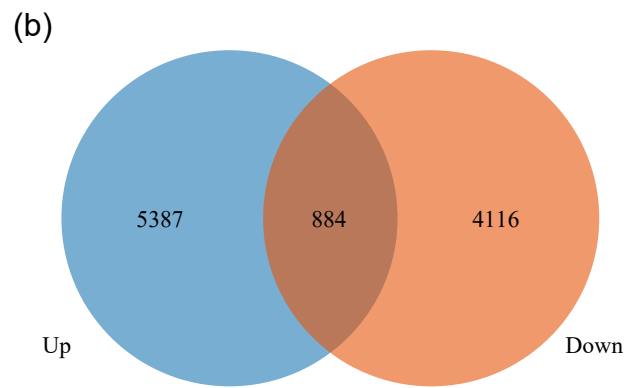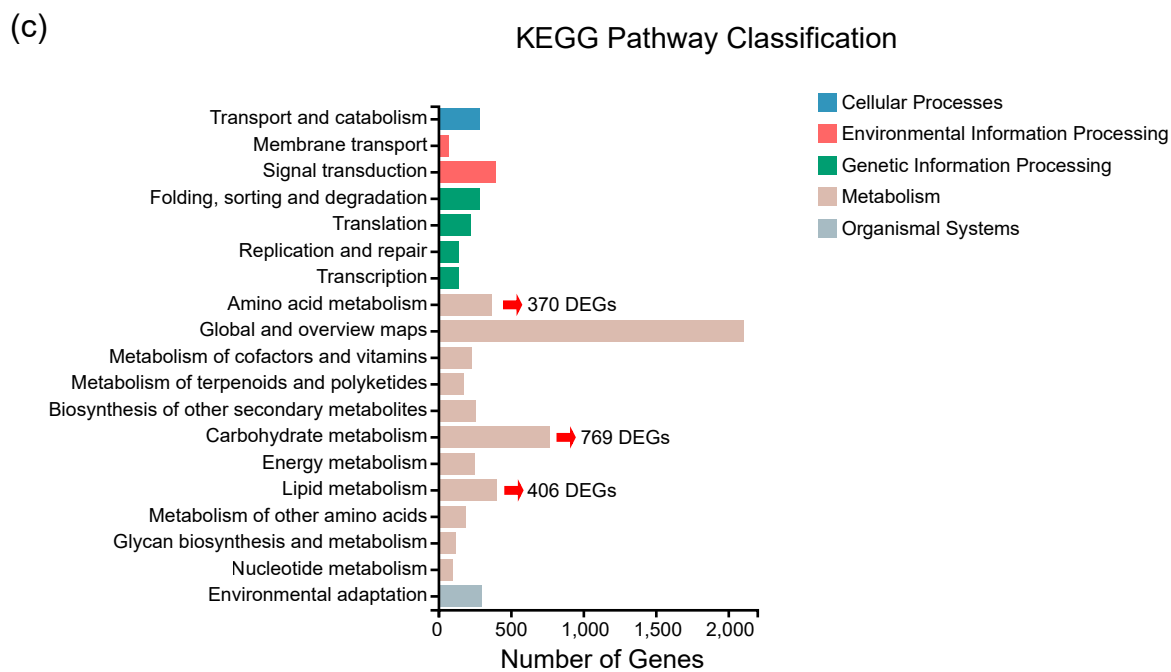

Supplement: Supplementary file 10 — Figure S10: Transcriptome analysis was performed using seeds of KO30‐1 and YH9326 collected at 35, 55, 75 and 85 days after flowering (DAF). (a) The number of DEGs between KO30‐1 and YH9326 at different seed developmental stage. (b) Venn diagram analysis of up‐ and down‐regulated differentially expressed genes. (c) Distribution of differentially expressed genes across KEGG pathway categories. [file PBI-24-4844-s006.pdf]
