# Supplementary material for: Mapping and Functional Characterization of Homologous Genes AhSUCA06 and AhSUCA16 Underlying Sucrose, Oil and Protein Contents in Peanut ( Arachis hypogaea L.)
Source: Plant Biotechnol J. 2026 Apr 18;24(8):4844–59. doi: 10.1111/pbi.70667 (PMC13387884; doi:10.1111/pbi.70667)
Supplement: Supplementary file 4 — Figure S4: Alignment of AhSUCA06 sequences amplified by PCR. (a) Alignment of AhSUCA06 sequences in JHT1 (Mu) and PI (WT); arahy.42CAD1 is the reference genome sequence. (b) Alignment of protein sequences encoded by AhSUCA06 in JHT1 (Mu) and PI (WT). The mutation site is indicated by a red box. [file PBI-24-4844-s009.pdf]

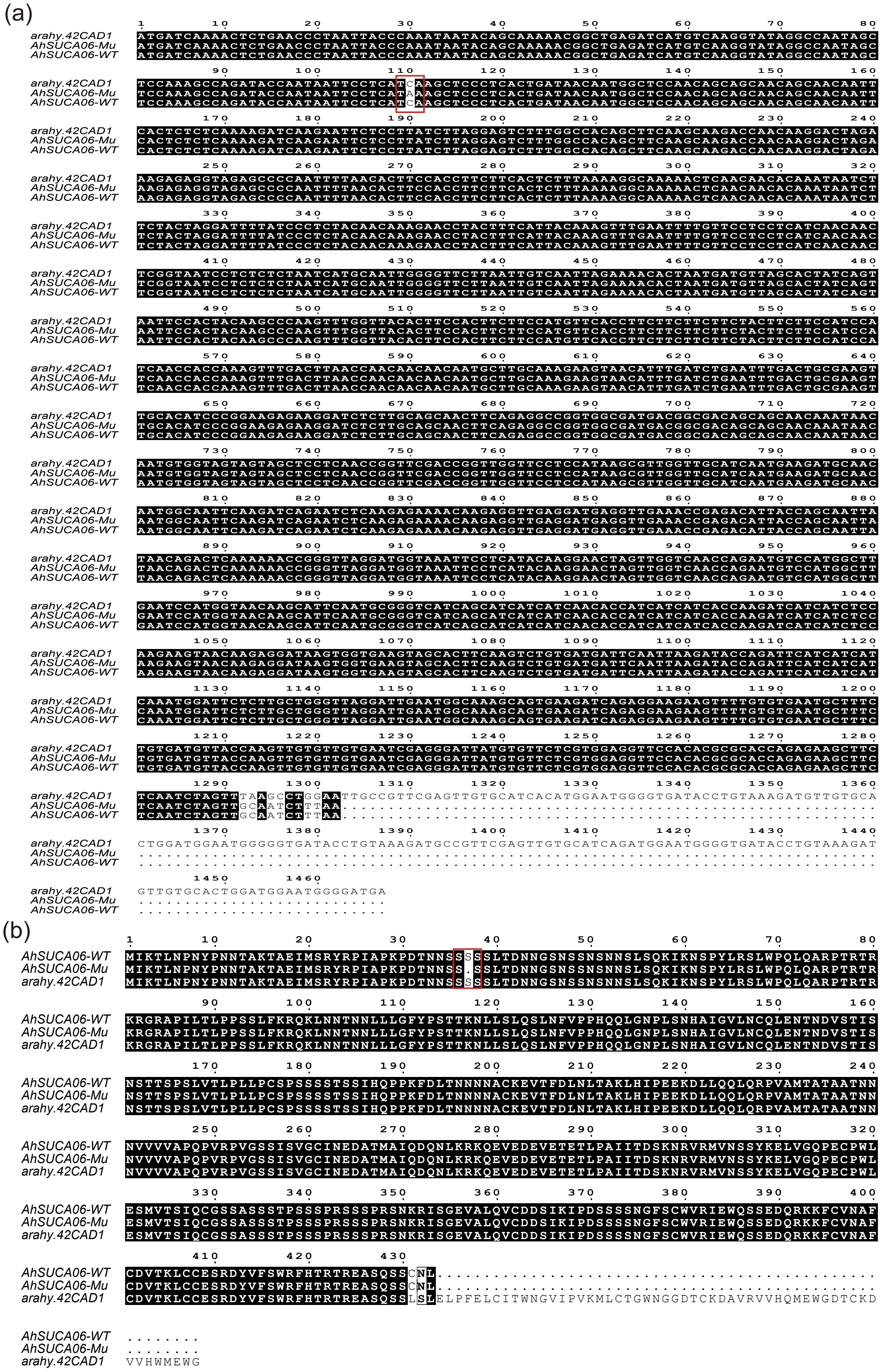
(a)
arahy.42CAD1
AhSUCA06-Mu
AhSUCA06-WT
(b)
AhSUCA06-WT
AhSUCA06-Mu
arahy.42CAD1
