# Supplementary material for: Mapping and Functional Characterization of Homologous Genes AhSUCA06 and AhSUCA16 Underlying Sucrose, Oil and Protein Contents in Peanut ( Arachis hypogaea L.)
Source: Plant Biotechnol J. 2026 Apr 18;24(8):4844–59. doi: 10.1111/pbi.70667 (PMC13387884; doi:10.1111/pbi.70667)
Supplement: Supplementary file 6 — Figure S6: Alignment of protein sequences encoded by AhSUCA16 in JHT1 (Mu) and PI (WT); arahy.3URM83 is the reference genome sequence. The InDel locus is indicated by a red box. [file PBI-24-4844-s005.pdf]

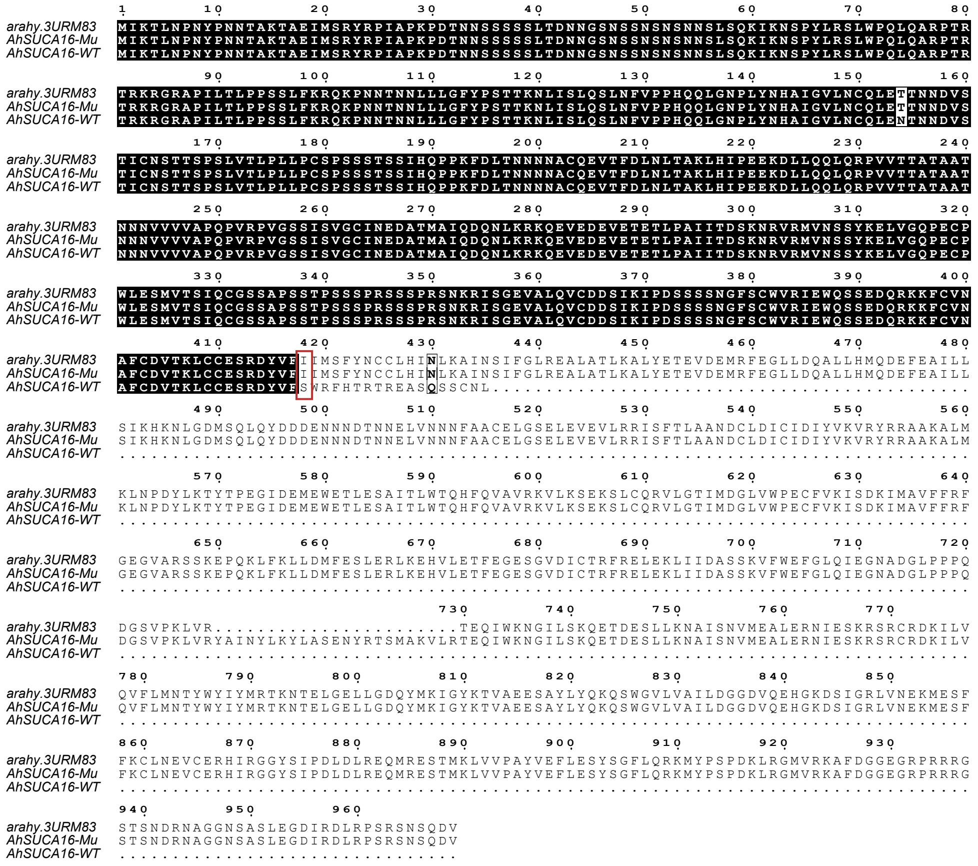
1 10 20 30 40 50 60 70 80
arahy.3URM83 MIKTLNPNYPNNTAKTAEIMSRYRPIAPKPDTNNSSSSSLTDNNGSNSSNSNSNNSLSQKIKNSPYLRSLWPQLQARPTR
AhSUCA16-Mu MIKTLNPNYPNNTAKTAEIMSRYRPIAPKPDTNNSSSSSLTDNNGSNSSNSNSNNSLSQKIKNSPYLRSLWPQLQARPTR
AhSUCA16-WT MIKTLNPNYPNNTAKTAEIMSRYRPIAPKPDTNNSSSSSLTDNNGSNSSNSNSNNSLSQKIKNSPYLRSLWPQLQARPTR
90 100 110 120 130 140 150 160
arahy.3URM83 TRKRGRAPILTLPPSSLFKRQKPNNTNNLLLGFYPSTTKNLISLQSLNFVPPHQQLGNPLYNHAIGVLNCQLETTNNDVS
AhSUCA16-Mu TRKRGRAPILTLPPSSLFKRQKPNNTNNLLLGFYPSTTKNLISLQSLNFVPPHQQLGNPLYNHAIGVLNCQLETTNNDVS
AhSUCA16-WT TRKRGRAPILTLPPSSLFKRQKPNNTNNLLLGFYPSTTKNLISLQSLNFVPPHQQLGNPLYNHAIGVLNCQLENTNNDVS
170 180 190 200 210 220 230 240
arahy.3URM83 TICNSTTSPSLVTLPLLPCSPSSSTSSIHQPPKFDLTNNNNACQEVTFDLNLTAKLHIPEEKDLLQQLQRPVVTTATAAT
AhSUCA16-Mu TICNSTTSPSLVTLPLLPCSPSSSTSSIHQPPKFDLTNNNNACQEVTFDLNLTAKLHIPEEKDLLQQLQRPVVTTATAAT
AhSUCA16-WT TICNSTTSPSLVTLPLLPCSPSSSTSSIHQPPKFDLTNNNNACQEVTFDLNLTAKLHIPEEKDLLQQLQRPVVTTATAAT
250 260 270 280 290 300 310 320
arahy.3URM83 NNNVVVVAPQPVRPVGSSISVGCINEDATMAIQDQNLKRKQEVEDEVETETLPAIITDSKNRVRMVNSSYKELVGQPECP
AhSUCA16-Mu NNNVVVVAPQPVRPVGSSISVGCINEDATMAIQDQNLKRKQEVEDEVETETLPAIITDSKNRVRMVNSSYKELVGQPECP
AhSUCA16-WT NNNVVVVAPQPVRPVGSSISVGCINEDATMAIQDQNLKRKQEVEDEVETETLPAIITDSKNRVRMVNSSYKELVGQPECP
330 340 350 360 370 380 390 400
arahy.3URM83 WLESMVTSIQCGSSAPSSTPSSSPRSSSPRSNKRISGEVALQVCDDSIKIPDSSSSNGFSCWVRIEWQSSEDQRKKFCVN
AhSUCA16-Mu WLESMVTSIQCGSSAPSSTPSSSPRSSSPRSNKRISGEVALQVCDDSIKIPDSSSSNGFSCWVRIEWQSSEDQRKKFCVN
AhSUCA16-WT WLESMVTSIQCGSSAPSSTPSSSPRSSSPRSNKRISGEVALQVCDDSIKIPDSSSSNGFSCWVRIEWQSSEDQRKKFCVN
410 420 430 440 450 460 470 480
arahy.3URM83 AFCDVTKLCCESRDYVFIIMSFYNCCLHINLKAINSIFGLREALATLKALYETEVDEMRFEGLLDQALLHMQDEFEAILL
AhSUCA16-Mu AFCDVTKLCCESRDYVFIIMSFYNCCLHINLKAINSIFGLREALATLKALYETEVDEMRFEGLLDQALLHMQDEFEAILL
AhSUCA16-WT AFCDVTKLCCESRDYVFSWRFHTRTREASQSSCNL...
490 500 510 520 530 540 550 560
arahy.3URM83 SIKHKNLGDMSQLQYDDDENNNDTNNELVNNNFAACELGSELEVEVLRRISFTLAANDCLDICIDIYVKVRYRRAAKALM
AhSUCA16-Mu SIKHKNLGDMSQLQYDDDENNNDTNNELVNNNFAACELGSELEVEVLRRISFTLAANDCLDICIDIYVKVRYRRAAKALM
AhSUCA16-WT ....
570 580 590 600 610 620 630 640
arahy.3URM83 KLNPDYLKTYTPEGIDEMEWETLESAITLWTQHFQVAVRKVLKSEKSLCQRVLGTIMDGLVWPECFVKISDKIMAVFFRF
AhSUCA16-Mu KLNPDYLKTYTPEGIDEMEWETLESAITLWTQHFQVAVRKVLKSEKSLCQRVLGTIMDGLVWPECFVKISDKIMAVFFRF
AhSUCA16-WT ....
650 660 670 680 690 700 710 720
arahy.3URM83 GEGVARSSKEPQKLFKLLDMFESLERLKEHVLETFEGESGVDICTRFRELEKLIIDASSKVFWEFGLQIEGNADGLPPPQ
AhSUCA16-Mu GEGVARSSKEPQKLFKLLDMFESLERLKEHVLETFEGESGVDICTRFRELEKLIIDASSKVFWEFGLQIEGNADGLPPPQ
AhSUCA16-WT ....
730 740 750 760 770
arahy.3URM83 DGSVPKLVR.......................TEQIWKNGILSKQETDESLLKNAISNVMEALERNIESKRSRCRDKILV
AhSUCA16-Mu DGSVPKLVRYAINYLKYLASENYRTSMAKVLRTEQIWKNGILSKQETDESLLKNAISNVMEALERNIESKRSRCRDKILV
AhSUCA16-WT ....
780 790 800 810 820 830 840 850
arahy.3URM83 QVFLMNTYWYIYMRTKNTELGELLGDQYMKIGYKTVAEESAYLYQKQSWGVLVAILDGGDVQEHGKDSIGRLVNEKMESF
AhSUCA16-Mu QVFLMNTYWYIYMRTKNTELGELLGDQYMKIGYKTVAEESAYLYQKQSWGVLVAILDGGDVQEHGKDSIGRLVNEKMESF
AhSUCA16-WT ....
860 870 880 890 900 910 920 930
arahy.3URM83 FKCLNEVCERHIRGGYSIPDLDLREQMRESTMKLVVPAYVEFLESYSGFLQRKMYPSPDKLRGMVRKAFDGGEGRPRRRG
AhSUCA16-Mu FKCLNEVCERHIRGGYSIPDLDLREQMRESTMKLVVPAYVEFLESYSGFLQRKMYPSPDKLRGMVRKAFDGGEGRPRRRG
AhSUCA16-WT ....
940 950 960
arahy.3URM83 STSNDRNAGGNSASLEGDIRDLRPSRSNSQDV
AhSUCA16-Mu STSNDRNAGGNSASLEGDIRDLRPSRSNSQDV
AhSUCA16-WT ....
